# Potential zoonotic pathogens hosted by endangered bonobos

**DOI:** 10.1038/s41598-021-85849-4

**Published:** 2021-03-18

**Authors:** Hacène Medkour, Sergei Castaneda, Inestin Amona, Florence Fenollar, Claudine André, Raphaël Belais, Paulin Mungongo, Jean-Jacques Muyembé-Tamfum, Anthony Levasseur, Didier Raoult, Bernard Davoust, Oleg Mediannikov

**Affiliations:** 1 Aix Marseille Univ, IRD, AP-HM, MEPHI, IHU-Méditerranée Infection, Marseille, France; 2 IHU-Méditerranée Infection, Marseille, France; 3 Aix Marseille Univ, IRD, AP-HM, SSA, VITROME, IHU-Méditerranée Infection, Marseille, France; 4 Les Amis des Bonobos du Congo, Kinshasa, Democratic Republic of the Congo; 5 National Institute of Biomedical Research (INRB), Kinshasa, Democratic Republic of the Congo

**Keywords:** Microbiology, Molecular biology, Infectious diseases

## Abstract

Few publications, often limited to one specific pathogen, have studied bonobos (*Pan paniscus*), our closest living relatives, as possible reservoirs of certain human infectious agents. Here, 91 stool samples from semicaptive bonobos and bonobos reintroduced in the wild, in the Democratic Republic of the Congo, were screened for different infectious agents: viruses, bacteria and parasites. We showed the presence of potentially zoonotic viral, bacterial or parasitic agents in stool samples, sometimes coinfecting the same individuals. A high prevalence of *Human mastadenoviruses* (HAdV-C, HAdV-B, HAdV-E) was observed. Encephalomyocarditis viruses were identified in semicaptive bonobos, although identified genotypes were different from those identified in the previous fatal myocarditis epidemic at the same site in 2009. Non-*pallidum Treponema* spp*.* including symbiotic *T. succinifaciens*, *T. berlinense* and several potential new species with unknown pathogenicity were identified. We detected DNA of non-*tuberculosis Mycobacterium* spp., *Acinetobacter* spp., *Salmonella* spp. as well as pathogenic *Leptospira interrogans*. Zoonotic parasites such as *Taenia solium* and *Strongyloides stercoralis* were predominantly present in wild bonobos, while *Giardia lamblia* was found only in bonobos in contact with humans, suggesting a possible exchange. One third of bonobos carried *Oesophagostomum* spp*.*, particularly zoonotic *O. stephanostomum* and *O. bifurcum*-like species, as well as other uncharacterized Nematoda. *Trypanosoma theileri* has been identified in semicaptive bonobos. Pathogens typically known to be transmitted sexually were not identified. We present here the results of a reasonably-sized screening study detecting DNA/RNA sequence evidence of potentially pathogenic viruses and microorganisms in bonobo based on a noninvasive sampling method (feces) and focused PCR diagnostics.

## Introduction

Human-animal-environmental interactions play a major role in understanding the spread of infectious agents that are pathogenic to humans^1^, and these interactions were the origin of the emergence of the "One Health" concept. To understand these interactions, samples from apes containing genetic material are required in order to conduct relevant studies and to assess the health status of a given population^2,3^. This was the case when gorillas and common chimpanzees^4^ were discovered to be the origin of devastating human pathologies such as HIV^5^, malaria^6,7^ and Ebola^8^. Today, the habitats of great apes are affected by extensive agriculture, mining, deforestation and the emission of toxic substances such as mercury and cyanide^9^. This leads to a decrease in the habitat area of primate populations and their population density^10^. In addition, there is continued poaching by humans. As they are genetically extremely close to humans and share the same environment, nonhuman primates (NHPs) promote the exchange and dispersal of pathogens with humans^11–13^. It is therefore essential to identify the full spectrum of microorganisms hosted by these primates to assess common interests. Discovering their natural cycles, virulence and viability are required to better understand infectious diseases in primates, implement control strategies and, probably, to predict possible spill-over episodes^14,15^. However, invasive methods to collect tissue samples from primates are not very feasible. Regulations and restrictions are in place to protect these animals from any potential damage.

The bonobo (*Pan paniscus*), discovered in 1929, is, along with the common chimpanzee (*Pan troglodytes*), the closest genetic relative of humans^16^. It is among the most endangered species according to the International Union for Conservation of Nature^17^. Bonobo populations are endemic only in the central lowland basin of equatorial Africa, south of the Congo River, in the Democratic Republic of the Congo (DRC). Dispersed in a fragmented manner along the river and its tributaries, gene flow is limited by these major environmental barriers. Poaching is the main threat these populations face, particularly due to the civil war that has taken place in the country. Population migration, habitat alteration through agricultural and commercial practices, and disruption of the education and health systems that replaced the traditional vision of indigenous bonobo protection are the main factors that are likely to reduce their populations in the coming decades^17^. A few publications have studied bonobos as possible reservoirs of certain pathogens. Each study was dedicated to the search for a specific pathogen (Table 1).

**Table 1:** Summary of previous studies conducted on bonobo (*Pan paniscus*) according to the size and nature of the samples used.

Infectious agents	Tests	Size and nature of samples
**Viruses**		
Human respiratory syncytial virus^18^	PCR and sequence fragments	8 carcasses of wild bonobos
Simian T-cell lymphotropic virus (STLV)^19^	PCR and sequence fragments	633 fecal samples from wild-living bonobos
*Herpesviridae*^20^	PCR and sequence fragments	21 blood samples from captive bonobos
Papillomavirus^21^	Histopathology, immunohistochemistry, PCR and full genome sequence	Tissue samples from a lesion in the oral cavity of a captive bonobo
Hepatitis E^22^	Serological cross reactivity (ELISA)	25 sera from captive bonobos
Adenovirus^23^	Virus isolation and full genome sequences	Number of fecal samples not specified of captive bonobos
Adenovirus^24^	PCR and sequence fragments	84 fecal samples from semicaptive bonobos
Encephalomyocarditis virus^25^	Histopathology, immunohistochemistry and PCR and sequence fragments	2 carcasses of semicaptive bonobos
Cytomegalovirus^26^	PCR and sequence fragments	33 fecal samples from semicaptive bonobos
Merkel cell polyomavirus^27^	PCR and full genome sequences	26 fecal samples from semicaptive bonobos
**Bacteria**		
*Streptococcus pneumoniae*^18^	PCR and sequence fragments	8 carcasses of wild bonobos
*Shigella* spp.^28^	Macroscopy	34 bonobos, articular surface
**Protozoa**		
*Plasmodium falciparum, P. malariae*^29^	PCR and sequence fragments	42 bonobo blood samples from Lola Ya sanctuary
*Plasmodium* (Laverania) *lomamiensis*^30^	PCR and sequences fragments	1556 fecal samples from wild bonobos
*Balantidium coli*^31^	Sheather's flotation and merthiolate-iodine-formaldehyde sedimentation	23 fecal samples from wild bonobos

However, the number of samples explored varies; studied animals lived in captivity (in most cases) far from their original distribution area in DRC. In addition, studies involving a larger number of samples concentrated on the search for one pathogen and did not provide information in terms of the potential of bonobos to be a reservoir for various microorganisms. Here, we intend to contribute to a better understanding of the role of bonobos as reservoirs/hosts of human pathogens by exploring the spectrum of associated pathogenic microorganisms using a noninvasive method. We looked for a wide range of zoonotic viral, bacterial, and parasitic agents, based on the available literature. We also compared two bonobo populations: bonobos from an orphan bonobo sanctuary, Lola Ya Bonobo (living in semicaptivity), and bonobos reintroduced in a natural reserve living in the wild (wild bonobos), Ekolo Ya Bonobo.

## Results

For screening purposes, we used whole DNA/RNA extracted from stool samples diluted in DNAse/RNAse-free water at 1:10. Indeed, pure DNA/RNA extracts from stool samples may contain a considerable amount of polymerase inhibitors^32^. The choice to use the dilution at 1:10 is therefore explained by the fact that we have sought to limit the polymerase inhibitors while having a sufficient DNA/RNA quantity^33^. Dilution at 1:100 would have allowed us to further limit the amount of inhibitor but with less DNA/RNA available. Nevertheless, confirmation of positive results was made using two other samples: pure DNA/RNA extracts and dilution at 1:100. The prevalence was calculated based on the results of the screening by qPCR.

### Viruses

**Figure 1:**
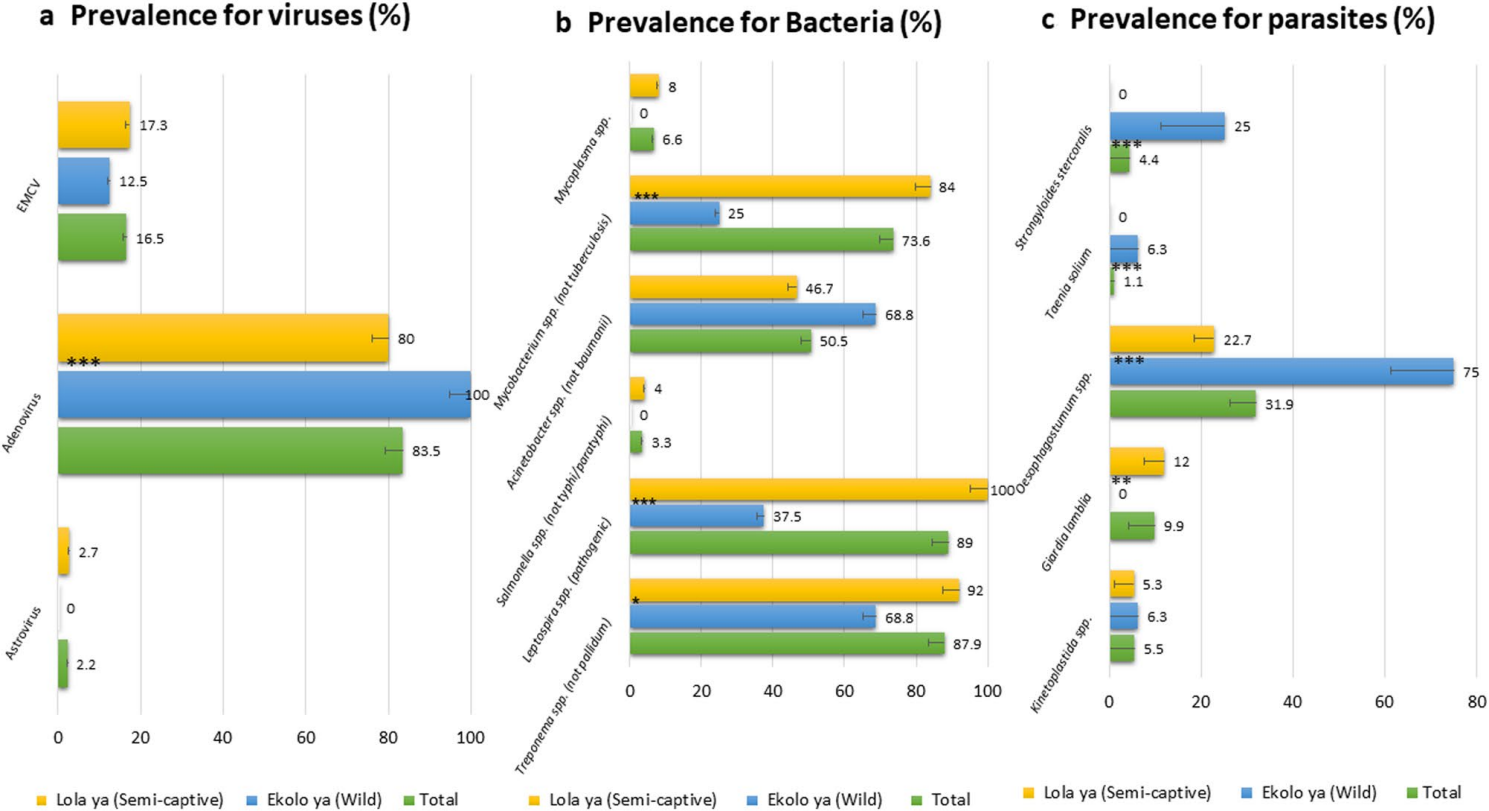
Large screening results; (**a**) Prevalence of viruses; (**b**) Prevalence of Bacteria; (**c**) Prevalence of parasites. ***Significative difference between Lola and Ekolo with p. value < 0.0001. **p. value < 0.03. *p. value < 0.05. Bars represent the error bars for percentages, they are showed by the lower half part of bars.

Among 15 viruses screened, three groups have been identified, namely, Astroviruses, Encephalomyocarditis virus (ECMV) and Adenoviruses (AdVs), in bonobo stool samples collected from the two sites in DRC (Fig. 1a).

Astrovirus was only found in 2.2% (2/91) of samples from the semicaptive bonobos of Lola Ya Bonobo (Lola). The prevalence of ECMVs was 16.5% (15/91) including 12.5% (2/16) from wild bonobos of Ekolo Ya Bonobo (Ekolo) and 17.3% (13/75) from Lola. A high prevalence of AdVs was detected, 83.5% (76/91), and all samples from Ekolo were positive 100% (16/16) *versus* 80% (60/75) positive in Lola (Z-test; P < 0.000001). All samples were negative for Sarbecoviruses including SARS-CoV-2, Enteroviruses, Hepatitis A and E viruses, Noroviruses, Parechoviruses, Poxviruses, Rotaviruses, SIV and HPV.

**Figure 2:**
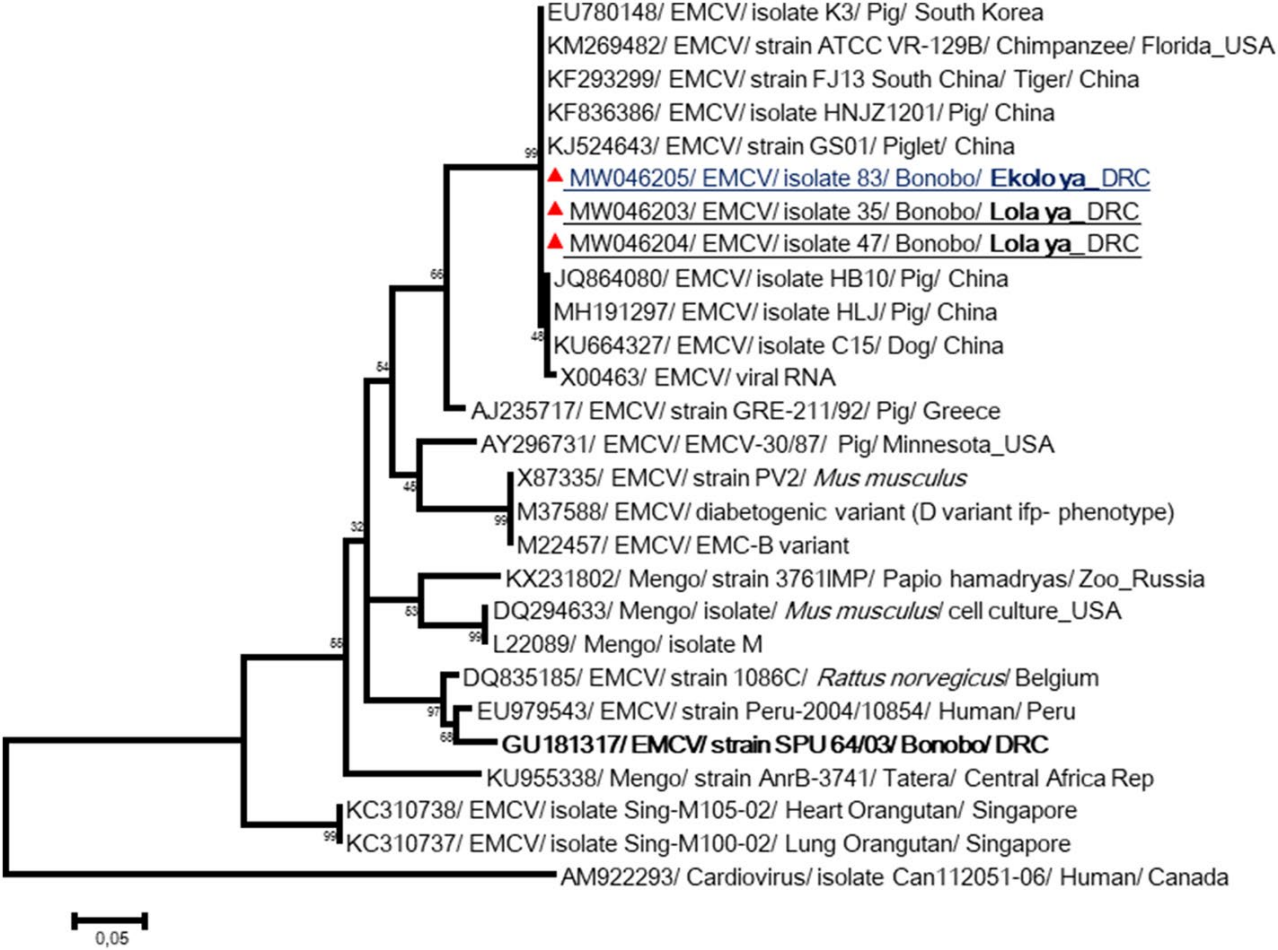
Phylogenetic tree for the partial sequence 3D of the Encephalomyocarditis viruses. The evolutionary history was inferred by using the Maximum Likelihood method based on the Tamura 3-parameter model. Sequences are identified as follows: accession number/virus/strain/host/country. Obtained sequences on samples from wild or semi-captive bonobos were closely identical to each other and to the available sequences in GenBank detected on other mammals (chimpanzee, pigs, tiger, rodents and dog). By contrast, they were distinct from the strain SPU64/03 (in bold), responsible of the fatal epizooty in Lola^25^. The tree is drawn to scale, with branch lengths measured in the number of substitutions per site. The analysis involved 27 nucleotide sequences. All positions containing gaps and missing data were eliminated. There were a total of 228 positions in the final dataset. Evolutionary analyses were conducted in MEGA7^61^.

The primers F1 and P1 were designed to amplify a part of the viral polymerase gene (*3D* gene) of EMCVs. Phylogenic analysis of the obtained sequences showed an identical strain in wild and semicaptive bonobos, and this was very similar to the ECMV Strain ATCC VR-129B (KM269482) isolated from a captive chimpanzee from Florida. Additionally, this strain showed > 99% similarity with other strains detected in pigs, tiger and a dog from China (Fig. 2).

**Figure 3:**
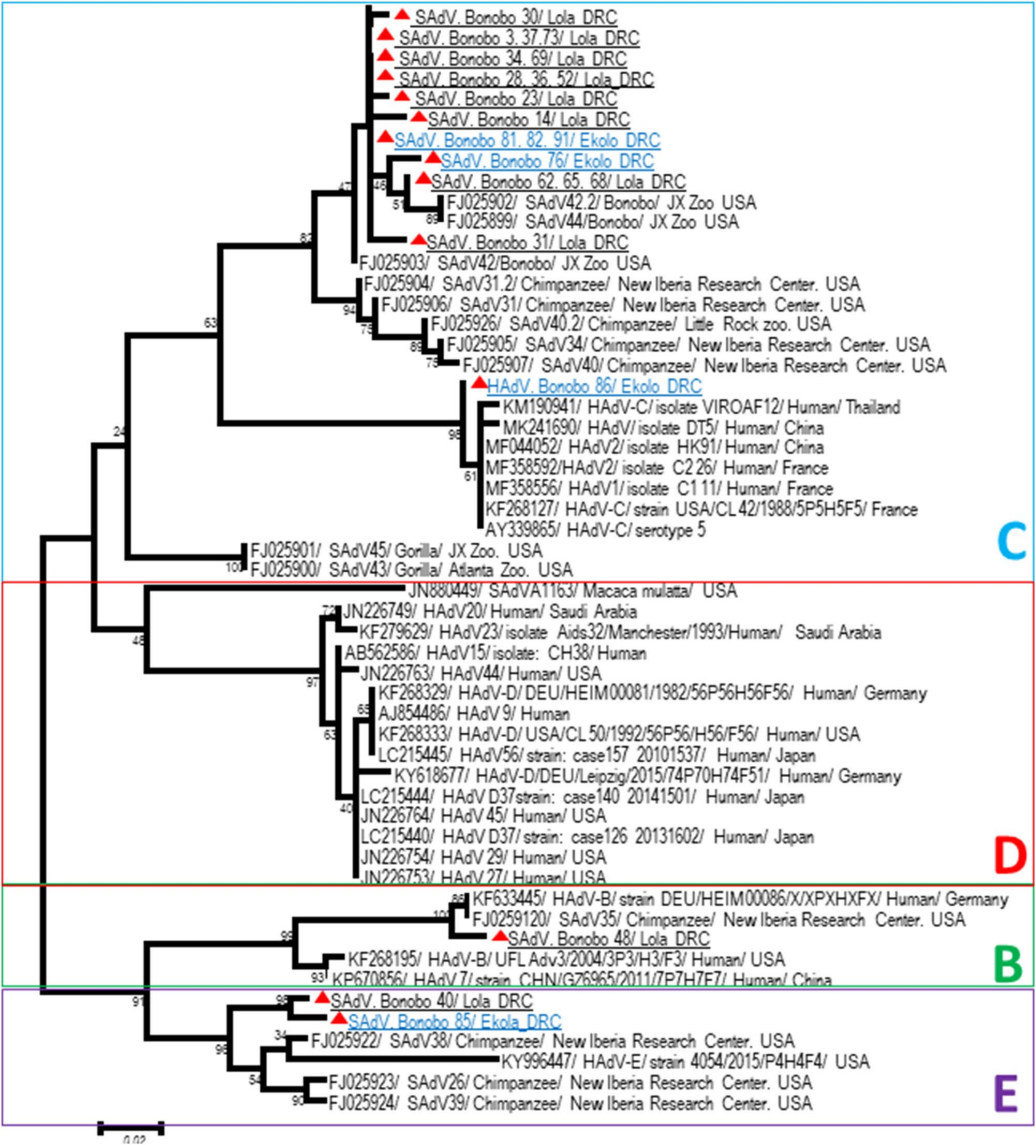
Phylogenetic analysis of Adenoviruses**.** The evolutionary history, based on 250 bp of *DNA PoL* gene, was inferred using the Neighbor-Joining method. Adenoviruses obtained sequences here were different in each other and with those available in GenBank database. Sequences are identified as follows: accession number/virus/strain/host/country.A similarity rate of 92 to 98% was obtained when comparing the obtained sequences from bonobos in Lola to reference strain of *Simian adenovirus* 42.1 (FJ025903) within HAdV-C species isolated from the gut of bonobo from Jacksonville zoo, USA^23^. Adenovirus strain (Bonobo 86) detected in Ekolo was 93% identity with a *Human adenovirus* sp. isolate DT5 (MK241690) isolated in a human from China. This suggests a possible “jump” of human strain to bonobos. Other species (HAdV-B and HAdV-E) were detected Lola and/or Ekola bonobos. The differences in the composition bias among sequences were considered in evolutionary comparisons. The analysis involved 39 nucleotide sequences. All positions containing gaps and missing data were eliminated. There were a total of 213 positions in the final dataset. Evolutionary analyses were conducted in MEGA7^61^.

Based on the DNA polymerase gene of AdVs, and by optimization of a nested-PCR protocol using the outer/inner primers previously described^23^, the obtained sequences in the present study were different from each other and from the available AdV sequences deposited in GenBank. Ten sequences (eight from Lola and two from Ekolo) were almost identical to each other and showed high similarity (92 to 98%) with SAdV-42.1 (FJ025903) and SAdV-42.2 (FJ025902) isolated from bonobos’ guts at Jacksonville Zoo, USA, while one AdV sequence detected in Ekolo (Bonobo86) shared only 93% identity with its closest relative *Human adenovirus* sp. isolate DT5 (MK241690) from a Chinese man. One sequence obtained from Lola Ya Bonobo was similar to SAdV-35 (FJ0259120) isolated from a chimpanzee from New Iberia Research Center, USA, and a strain (KF633445) detected in a human from Germany, within the HAdV-B species. In addition, two sequences, one from a semicaptive bonobo and another from a wild one, were similar to each other and close to HAdV-E detected in chimpanzees (SAdV-26 and SAdV-39) from New Iberia Research Center and a man from the USA (Fig. 3, Table 2).

**Table 2:** Generated sequences in this study and their identification.

Agents	Used primers	Length and Tm	Obtained sequences	Amplified fragment	New sequence ID	Best blast hits (genebank acc number)
EMCVs	P1-CCCTACCTCACGGAATGGGGCAAAG F1-TTATWATTAGGGCIGGYTTG F2-CTAGCAAAGACAGGRTAYAA R2-ACGRAAIGGGGCAAAGAG	Nested PCR: P1/F1 (400 bp) at 55 °C; then F2/R2 (250 bp) at 55 °C	3	250 bp of *3D*	MW046203–MW046205	> 99% with ECMV Strain ATCC VR-129B (KM269482)
Adenoviruses	Fw Outer-TGATGCGYTTCTTACCTYTGGTYTCCATGAG Rw Outer-AGTTYTACATGCTGGGCTCTTACCG Fw inner-GTGACAAAGAGGCTGTC-CGTGTCCCCGTA Rw inner- TCACGTGGCCTACACTTACA-AGCCAATCAC	Nested PCR: outer primers (≈ 1400 bp) at 58 °C, then Inner primers (≈ 250 bp) at 55 °C	10	250 bp of *DNA pol*	–	92 to 98% with SAdV-42.1 (FJ025903) and SAdV-42.2 (FJ025902) belonged to AdV-C
			1			93% with HAdV sp. isolate DT5 (MK241690) belonged to AdV-C
			1			SAdV-35 (FJ0259120) belonged to AdV-B
			2			SAdV-26 (FJ025923) and SAdV-39 (FJ025924) belonged to AdV-E
*Leptospira* spp.	F- TAGGAAATTGCGCAGCTACA R- GCATCGAGAGGAATTAACATCA	F/R (520 bp), 53 °C	5	460–520 bp of *LipL41*	MW067650–MW067654	> 99% with *L. interrogans* serovar Grippotyphosa (JQ690557)
*Treponema* spp.	F1-GGGAGTGAGACTGCGIGCG R2- GGTGTCASCMCCTATACGTCYCAT	F1/R2 (900 bp), 57 °C	5	787–1039 bp of *23S*	MT257118–MT257135	> 99.5% with *T. succinifaciens* strain 609 (NR076867) and > 97.5% with *Treponema* spp. from other African NHPs
			3			95–98% with *T. succinifaciens* (NR076867)
			1			93% with *T. succinifaciens* DSM 2489 (CP002631)
			3			83% with *T. succinifaciens* DSM 2489 and *T. brennaborense* DSM 12,168 (CP002696)
			7			78–88% with *T. brennaborense* and 81–99% with *T. berlinense* (FUXC01000026)
*Oesophagostumum* spp.	Fwd.18S.631-TCGTCATTGCTGCGGTTAAA Rwd.18S.1825r- GGTTCAAGCCACTGCGATTAA	1127–1155 bp, 54 °C	1	1100 bp of *18S*	MT89 0583	99.5% and 99.4% with *O. aculeatum* (AB677956) and *O. muntiacum* NSMT:As4470 (LC415112)
	NC1-TTAGTTTCTTTTCCTCCGCT NC2-ACGTCTGGTTCAGGGTTGTT OesophITS2-21TGTRACACTGTTTGTCGAAC	Hemi-nested PCR: NC1/ NC2 (280–400 bp), 50 °C; NC2/ OesophITS2-21 (260 bp), 55 °C	27	268–307 bp of ITS2 region	MW040123–MW040151	> 99% with *O. stephanostomum* (KR149651)
			2			97% with *O. bifurcum* (MT184890) and with *O. cf. aculeatum* (AB586134)
*Kinetoplastida* sp.	F720-GTTAAAGGGTTCGTAGTTGAA R1425- GACTACAATGGTCTCTAATCA	F720/R1425 (≈ 750 bp), 50 °C	3	475–522 bp of *18S*	MT886281–MT886283	> 99% identity with *Trypanosoma theleiri* (KR024688)
			1		MT886284	> 99% *Bodo saltans* (MH614643)

### Bacteria

Screening detected 27 bacterial agents listed in Table 3, including principal zoonotic agents. Pathogenic *Leptospira* spp. have been detected in 89% (81/91) of bonobos, and the prevalence was higher in Lola than in Ekolo (100% vs 37%, P < 0.001). *Mycobacterium* spp. (non-*tuberculosis*) have been detected in 73.6% (67/91), and again, the prevalence was higher in Lola (84%) *vs* (25%) Ekolo (Z-test; P < 0.001). In addition, the highest prevalence (88%) was noted for *Treponema* spp. (non-*pallidum*), and animals from Lola were more frequently infected (92%) than bonobos from Ekolo (87%) (P = 0.049). *Acinetobacter* spp. (non-*baumanii*) DNA was found in 69% of bonobos with a prevalence almost equal at the two sites. Finally, *Salmonella* spp. (non-*typhi/paratyphi*) and *Mycoplasma* spp. were detected in 3.3% and 6.6% of bonobos, respectively; all of them were from Lola (Fig. 1b). DNA of *Bartonella, Borrelia, Chlamydia, Anaplasma, Wolbachia* and *Rickettsia* spp*., Rickettsia felis, Coxiella burnetii, Helicobacter pylori, Campylobacter* spp*., Mycobacterium tuberculosis, Acinetobacter baumannii, Salmonella paratyphi/typhi, Treponema pallidum, Tropheryma whipplei*, *Clostridium difficile, Neisseria gonorrhoeae, Atopobium vaginae, Gardnerella vaginalis, Listeria monocytogenes, Vibrio cholerae* and *Yersinia pestis* was not detected.

**Table 3:** Pathogens for which tests have been carried out.

Bacteria	Parasites	Viruses
*Acinetobacter* spp.	*Ancylostoma duodenale*	Adenovirus
*Acinetobacter baumannii*	*Ascaris lumbricoides*	Astrovirus
*Anaplasmataceae*	*Cryptosporidium parvum, C. hominis*	Encephalomyocarditis virus
*Bartonella* spp.	*Cyclospora cayetanensis*	Enterovirus
*Borrelia* spp.	*Entamoeba histolytica*	Hepatitis A virus
*Chlamydia* spp.	*Enterobius vermicularis*	Hepatitis E virus
*Coxiella burnetii*	*Filarioidea*	Norovirus
*Helicobacter pylori*	*Giardia lamblia*	Parechovirus
*Mycobacterium* spp.	Kinetoplastida	Poxvirus
*Mycoplasma* spp.	*Leishmania* spp.	Rotavirus
*M. genitalium*	*Loa loa*	Sapovirus
*Rickettsia* spp.	*Mansonella* spp.	Simian immunodeficiency virus (SIV)
*Rickettsia felis*	*Necator americanus*	Papillomavirus (HPV)
*Salmonella* spp.	Nematoda	Sarbecovirus
*Salmonella paratyphi/typhi*	*Plasmodium* spp.	Sars-Cov 2
*Staphylococcus aureus*	*Schistosoma mansoni*	Herpes simplex virus
*Treponema* spp.	*Strongyloides stercoralis*	
*Treponema pallidum*	*Taenia saginata*	
*Tropheryma whipplei*	*Taenia solium*	
*Vibrio cholerae*	*Toxoplasma gondii*	
*Wolbachia* spp.	*Trichuris trichiura*	
*Yersinia pestis*	*Physaloptera* spp.	
*Neisseria gonorrhoeae*	*Oesophagostomum* spp.	
*Atopobium vaginae*		
*Gardnerella vaginalis*		
*Listeria monocytogenes*		
*Leptospira* spp.		
*Clostridium difficile*		

**Figure 4:**
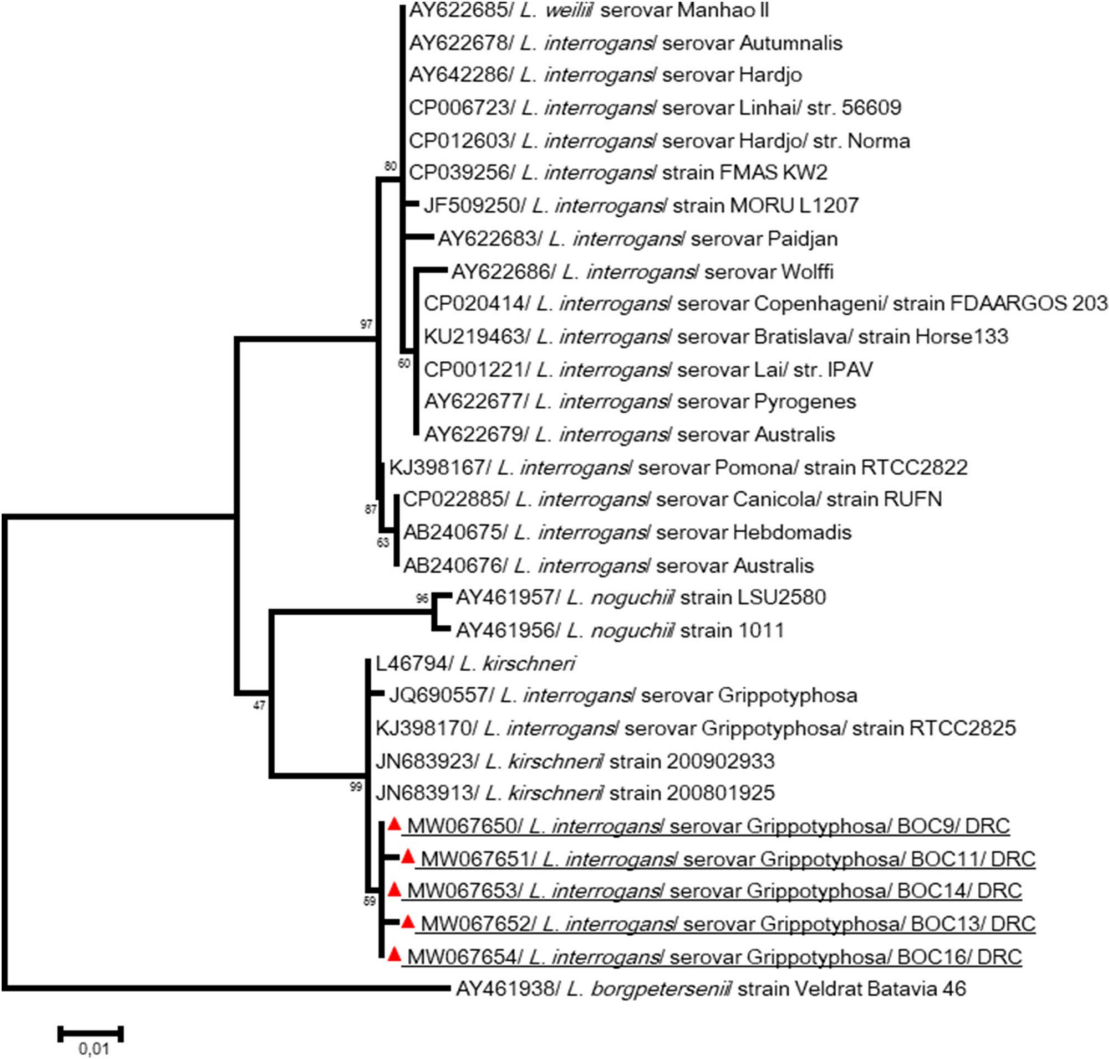
Phylogenetic analysis of *Leptospira* spp. from bonobo fecal samples. The evolutionary history, based on *LipL 41* partial gene, was inferred by using the Maximum Likelihood method based on the Tamura 3-parameter model. Initial treefor the heuristic search were obtained automatically by applying Neighbor-Join and BioNJ algorithms to a matrix of pairwise distances estimated using the Maximum Composite Likelihood (MCL) approach, and then selecting the topology with superior log likelihood value. Sequences are identified as follows: accession number/species/strain/host/country. Obtained sequences in the present study showed almost similarity in each other and with *Leptospira interrogans serovar Grippotyphosa* RTCC2825 (KJ398170). The analysis involved 31 nucleotide sequences. All positions containing gaps and missing data were eliminated. There were a total of 436 positions in the final dataset. Evolutionary analyses were conducted in MEGA7^61^.

*Leptospira* spp.-positive samples were amplified by PCRs targeting six genes: LipL 32, LipL 41, Adk, Icda, rrs 2 and sec-Y^34^. LipL 41 partial gene-positive 460–520-bp samples were sequenced. Five good quality sequences obtained from Lola were almost similar to each other, and they showed > 99% identity to highly pathogenic *Leptospira kirschneri* (previously called *L. interrogans* serovar *Grippotyphosa*) (JQ690557) (Fig. 4).

**Figure 5:**
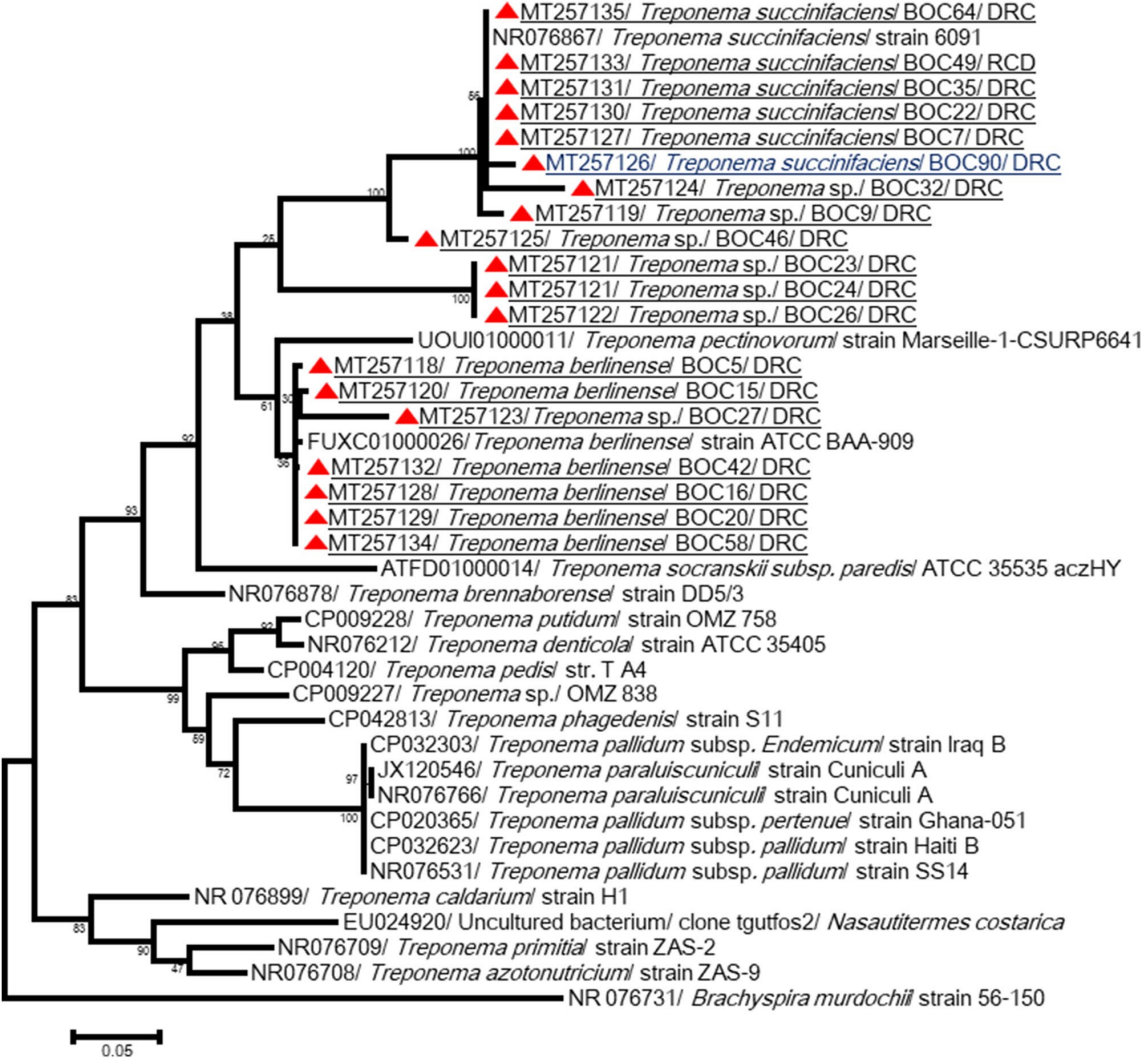
Molecular Phylogenetic analysis for *Treponema* spp. detected on bonobos from DRC. The evolutionary history, based on partial *23S rRNA* gene, was inferred by using the Maximum Likelihood method based on the Tamura 3-parameter model. Initial tree for the heuristic search were obtained automatically by applying Neighbor-Join and BioNJ algorithms to a matrix of pairwise distances estimated using the Maximum Composite Likelihood (MCL) approach, and then selecting the topology with superior log likelihood value. Sequences are identified as follows: accession number/species/strain/host/country. Sequences in this study are highlighted by black circle and underlined. Sequence wrote in bleu was obtained on a wild bonobo sample while all the others obtained on semi-captive bonobo samples. In addition, in bolt are the *Treponema* spp. sequences from African NHPs. The tree is drawn to scale, with branch lengths measured in the number of substitutions per site. The analysis involved 48 nucleotide sequences. All positions containing gaps and missing data were eliminated. There were a total of 721 positions in the final dataset. Evolutionary analyses were conducted in MEGA7^61^.

The 16S rRNA of *Treponema* spp. was amplified, but sequencing has not been successful, probably due to the nonspecificity of primers (multiple peaks superposition). The primer sets for the 23S rRNA gene were more specific and allowed a total of 19 sequences of 787–1039 bp, exhibiting 83–100% identity to each other, to be obtained. Five sequences obtained from samples from Lola showed > 99.5% identity with *T. succinifaciens* strain 609 (NR076867). The sequences also exhibited > 97.5% similarity with *Treponema* spp. detected in other African great apes and monkeys (Algerian macaque, hamadryas from Djibouti, Guinea baboon and a green monkey from Senegal, gorilla and human individuals from the Congo) (Medkour et al. submitted). Three others, including one sequence obtained from an Ekolo bonobo (MT257126), were close and showed 95–98% identity to *T. succinifaciens* (NR076867) as well as the sequences of NHPs described above. One other sequence (MT257125) forms a single branch and was 93% similar to *T. succinifaciens* DSM 2489 (CP002631). Three similar sequences constitute another branch, and they were 83% identical to *T. succinifaciens* DSM 2489 and *T. brennaborense* DSM 12,168 (CP002696). They were almost identical (98%) to *Treponema* spp. detected in the feces of a gorilla in the Congo and a macaque in Algeria. Finally, seven sequences were 94.5–100% identical to each other and clustered with other sequences from *Treponema* spp. detected in African NHPs, such as clone G06B (MT257098) from a gorilla in the Congo, clone RS18 (MT257249) from an Algerian macaque, and clone Bab3 (MT257103) and clone CH32 (MT257113) detected in a Senegalese baboon and chimpanzee, respectively. In addition, the sequences showed 78–88% similarity with the official strain *T. brennaborense* and 81–99% with *T. berlinense* (FUXC01000026) (Fig. 5, Table 2).

### Parasites

Samples were screened for the main zoonotic parasites (Table 3). Bonobos from Ekolo carried more parasites than in Lola, except for *Giardia lamblia* (9.9% of bonobos), which has been detected only in individuals living in semicaptivity (12% in Lola *versus* 0% in Ekolo; Z test; P = 0.03)*.* The prevalences (in Ekolo *versus* Lola; Z test P-value) were 4.4% for *Strongyloides stercoralis* (25% *versus* 0%; P < 0.001) including two samples detected as positive by qPCR-Nematoda, 1.1% for *Taenia solium* (6.3% *versus* 0%; P < 0.001), 15.4% for *Nematoda* spp. (31.3% *versus* 12%; P > 0.05) and 5.5% for *Kinetoplastida* spp. (6.3% *versus* 5.3%; P > 0.05) including 4.4% positive for *Trypanosoma* spp. (Fig. 1c).

Samples were also screened using specific qPCRs for *Filarioidea*, *Mansonella* spp., *Loa loa*, *Physaloptera* spp., *Ancylostoma duodenale, Ascaris lumbricoides, Cryptosporidium parvum/C. hominis, Cyclospora cayetanensis, Entamoeba histolytica, Enterobius vermicularis, Leishmania* spp*., Plasmodium* spp*., Piroplasmida* spp., *Necator americanus, Schistosoma mansoni, Toxoplasma gondii,* and *Trichuris trichiura*, and all of them were found to be negative.

**Figure 6:**
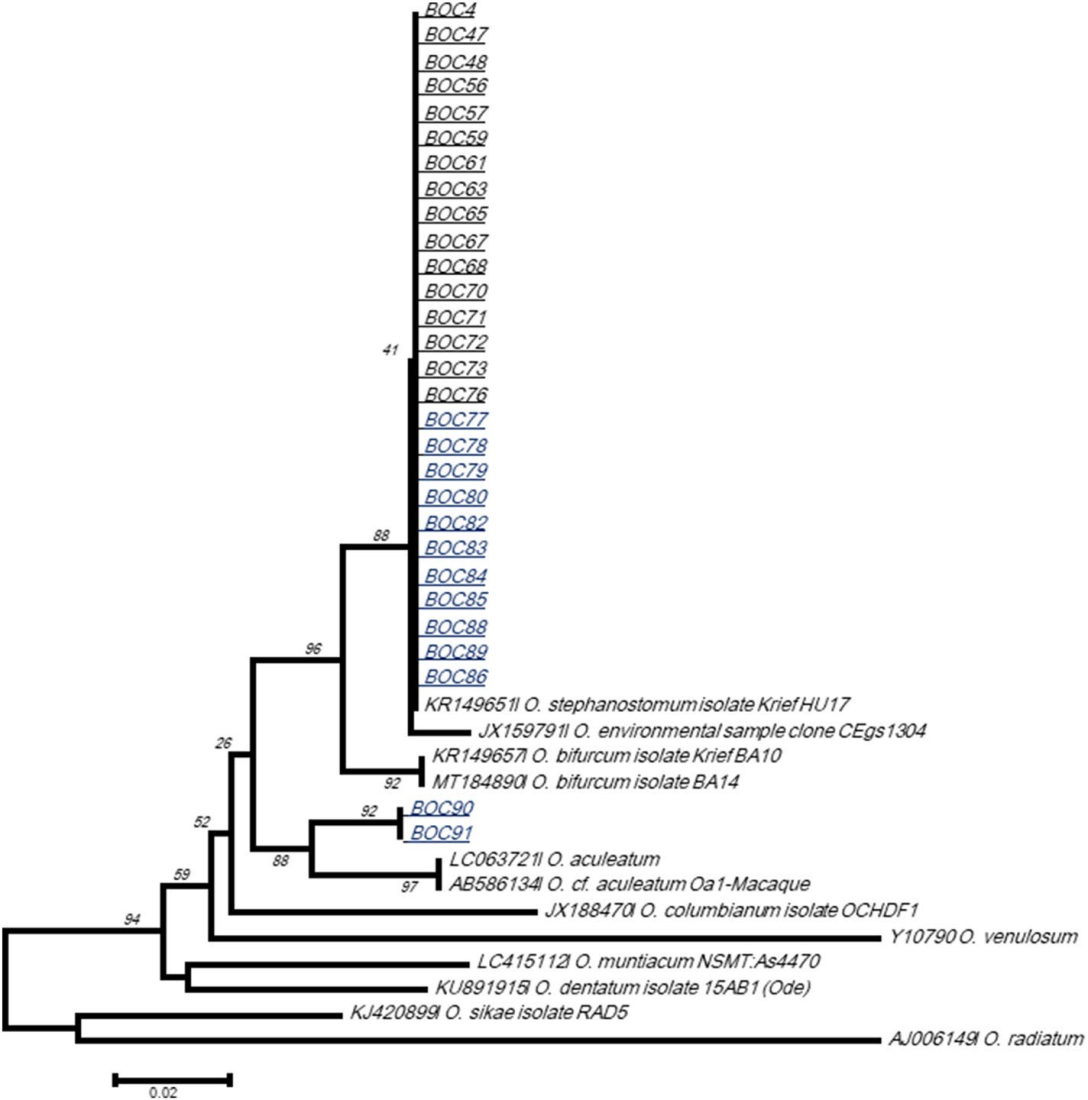
Phylogenetic analysis of *Oesophagostomum* based on ITS2 rDNA (260 bp) sequences. The evolutionary history was inferred using the Neighbor-Joining method. Sequences in this study are named “BOC”, those in black are from Lola and those in bleu are from Ekolo. The tree is drawn to scale, with branch lengths in the same units as those of the evolutionary distances used to infer the phylogenetic tree. The evolutionary distances were computed using the Tamura-Nei method and are in the units of the number of base substitutions per site. The differences in the composition bias among sequences were considered in evolutionary comparisons. The analysis involved 41 nucleotide sequences. All positions containing gaps and missing data were eliminated. Evolutionary analyses were conducted in MEGA7^61^.

To identify nematodes detected by pan-Nematoda qPCR, the partial Cox1 and 18S rRNA genes were successfully amplified. Possibly due to multiple coinfections by more than one Nematode species, electropherograms containing double peaks were difficult to analyze. However, for one sample from Ekolo, an 18S rRNA sequence of 1100 bp was obtained and showed 99.5% and 99.4% identity with *Oesophagostomum aculeatum* (AB677956) and O. *muntiacum* NSMT:As4470 (LC415112), respectively (Fig. S1). This sequence showed > 99.7% identity with *Oesophagostomum* isolates CC09 and CC37 (MT260066 and MT260068) detected in Barbary macaques from Algeria^35^. After that, all samples were screened using a nested PCR targeting the ITS2 region of *oesophagostumum* spp. and positive were sequenced. We found 31.9% of positive including 75% in Ekolo *versus* 22.7% in Lola (P < 0.0001). All obtained sequences, except two, were almost similar and showed > 99% with *O. stephanostomum* (KR149651). The two other sequences showed 97% identity with *O. bifurcum* (MT184890) and with *O. cf. aculeatum* (AB586134) (Fig. 6).

**Figure 7:**
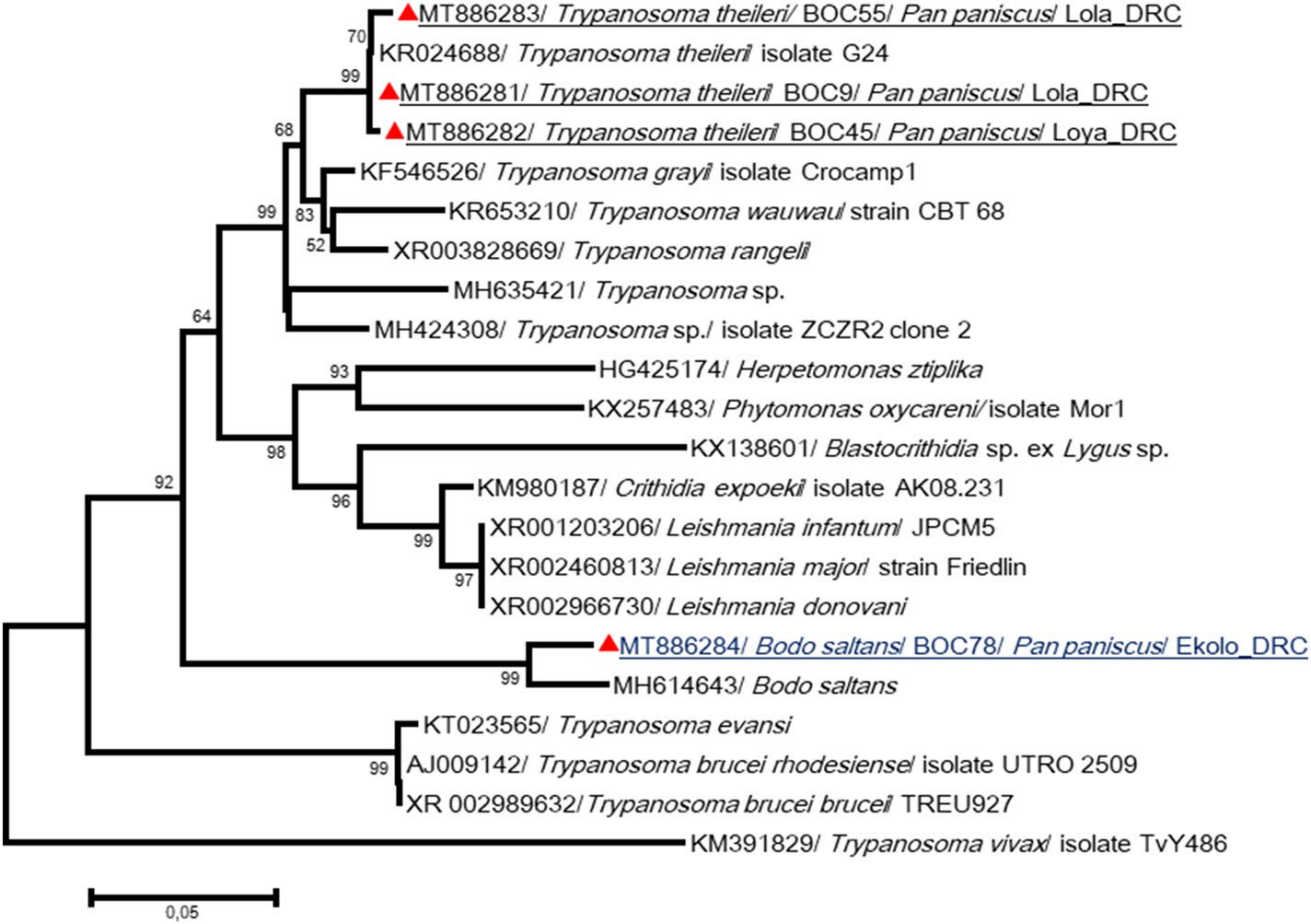
Phylogenetic analysis of *Kinetoplastida* spp. detected in this study. The evolutionary history, based on *18S rRNA* gene, was inferred using the Neighbor-Joining method. The obtained sequences here were compared to sequences of *Kinetoplastida* spp. available in GenBank. Sequences are identified as follows: accession number/species/strain/host/country. Sequences in Lola were almost identical and presented > 99% identity with *Trypanosoma theleiri* (KR024688), known as pathogen for ruminants. Whereas, in Ekolo, one other sequence was closely identical to *Bodo saltans* (MH614643)*,* a free living nonpathogenic kinetoplastid. The analysis involved 22 nucleotide sequences. All positions containing gaps and missing data were eliminated. There were a total of 483 positions in the final dataset. Evolutionary analyses were conducted in MEGA7^61^.

The 18S and 28S rRNA partial genes for *Kinetoplastida* spp. were also amplified. Three 18S rRNA sequences obtained from bonobos from Lola were almost identical to each other and showed > 99% identity with *Trypanosoma theleiri* (KR024688). In addition, in Ekolo, one other sequence was closely identical to *Bodo saltans* (MH614643) (Fig. 7, Table 2).

## Discussion

The objective of this study was to identify the spectrum of zoonotic bacteria, parasites and viruses in two populations of bonobos, one semicaptive (Lola) living every day in contact with humans and the other living in the wild (Ekolo), and to compare the pathogen distribution between the two sites. Ekolo Ya Bonobo is the only bonobo reintroduction site in the world. The first group led by the "female Alfa" Etumbe was reintroduced in 2008. The expansion of the protected area (Ekolo) will ensure sufficient space to continue to reintroduce bonobo groups rescued from the bushmeat trade and rehabilitated in Lola Ya Bonobo.

The use of stool samples, a noninvasive sampling technique, to study pathogens is very important. In addition to enteric pathogens that are logically easy to find in stool samples, this technique allows the identification of microorganisms usually residing in blood (malaria^6^, *Leishmania*^36^, filaria^35^, etc.) and in the urogenital system, such as *Leptospira* (this study). This sampling technique is absolutely noninvasive and easily authorized for NHPs. In this study, 91 bonobo stool samples were treated. This is the first large-scale systematic screening by PCR/RT-PCR of zoonotic pathogens in bonobos using fecal samples. Previous publications reported infectious agents found in various kinds of samples from bonobos including blood, stool, serum, secretion swab, urine, cadaver, etc. (Table 1).

Here, adenovirus carriage was very high in both wild and semicaptive bonobos, and great genetic diversity was highlighted. *Human mastadenovirus* C (HAdV-C) was present in bonobos cohabiting with humans and those released in the forest. These results reinforce the exchange between human and bonobo viruses, since we detected a HAdV-C strain in a bonobo very close to strains detected in humans (Fig. 3). In addition, the presence of AdVs in wild bonobos is evidence of the continuous circulation of adenoviruses in the bonobo population; we have identified the same viruses in individuals living in semicaptivity (in Lola) as well as those that were released in the wild 4 years ago. It is highly likely that bonobos released in Ekolo were already infected by these adenoviruses at that time, so it seems that we observed the persistence of HAdV-C in bonobos. This is not surprising because in humans, HAdV species C has the capacity to establish persistent infection in intestinal T lymphocytes of the digestive tract^37^. Furthermore, the presence of persistent and/or latent AdV infections in the gut of great apes, including bonobos, has been observed^23^ and should be considered in the design and interpretation of human and NHP studies (including vaccine development) with adenovirus vectors.

In our recent study on AdV circulation in African humans and NHPs, HAdV-B, HAdV-C, HAdV-D and HAdV-E were found in both humans and/or NHPs from the Congo. We demonstrated a possible jump of a human strain of HAdV-C to gorillas, and, vice versa, a gorilla strain of HAdV-C to humans sharing the same living area in the Congo^33^. HAdV-B members were detected in Lola and HAdV-E in Ekolo, and they were previously reported in captive bonobos from the Jacksonville Zoo, USA^23^. In a study of 800 fecal samples from wild African great apes and humans to investigate the evolutionary history and zoonotic potential of hominine HAdVs, HAdV-B and -E were frequently detected in wild gorillas (55%) and chimpanzees (25%), respectively. It was shown that HAdV-B circulating in humans are of zoonotic origin and have probably affected global human health for most of our species lifetime^24^. The finding in HAdVs in bonobo which have been reintroduced into the wild is very alarming and shows the risk of such reintroduction programs. In our study, the Lola Ya sanctuary followed the international recommendations for reintroduction^38^. In addition, because of the methodology, it could be possible that very divergent segments were not picked up by the primers, especially those of simian AdVs. Therefore, the nonidentified AdVs here could be SAdVs or AdVs from other species, which were not amplified by the applied primers. Furthermore, a DNA polymerase fragment was selected in order to be highly conserved. It is therefore no surprise that the fragment was found to be considerably conserved.

In 2009, a devastating epizooty due to ECMV in Lola led to fatal myocarditis in bonobos^25^. Here, EMCV genotypes detected in Lola and Ekolo bonobos were identical and close to other strains detected in mammals, including humans and chimpanzees. By contrast, they were very divergent to EMCV strain SPU 64/03 responsible for Lola bonobo mortality, which confirms the circulation of more than one EMCV strain in this area (Fig. 2). Highly divergent EMCVs were isolated from orangutans after fatal myocarditis in Singapore^39^. Several species of captive NHPs are susceptible to highly fatal EMCV myocarditis including the chimpanzee (*Pan troglodytes*), African green monkeys (*Chlorocebus*), squirrel monkeys (*Saimiri sciureus*), baboons (*Papio* spp.), macaques (*Macaca fascicularis* and *Macaca sylvanus*) and orangutan (*Pongo pygmaeus*)^25^. EMCV cases require particular attention. Taking into consideration the extremely high mortality due to viral encephalomyocarditis in apes and monkeys, especially those kept in captivity and, in general, the severe clinical picture, it seems to be urgent to accumulate epidemiological data concerning the circulation of these viruses. Virtually nothing is known about the epidemiology of these viruses in simians, for example, their transmission, origins, reservoirs or possibility of infecting humans. Bonobo dormitories were exposed to rates that can be the origin of this infection. Rodents were suspected as reservoirs and diagnosed epidemics in African wildlife. A study showed a striking temporal correlation between the occurrence of a population explosion (as evidenced by markedly increased catch rates per trap-night) and a surge in prevalence of antibody to EMCVs in rodents, and the occurrence of the outbreak of disease in elephants in South Africa^40^.

Bonobos host a variety of *Treponema* species. We identified at least seven genomospecies of *Treponema* in their feces including *T. succinifaciens*, which was identified in all bonobos, as well as previously reported for African gorilla, chimpanzee, green monkey, Guinea baboon, hamadryas, macaque and the human gut (Medkour et al. submitted). It seems that this species is a part of the gut microbiota, but its role is poorly understood. We also showed the existence of potential new species with unknown pathogenicity (Fig. 5). Spirochaetes has been reported in the gut microbiota of NHPs^41^. *Treponema* species have been detected in ancient^42^ and traditional rural human populations^43,44^. All traditional rural populations were enriched for *T. succinifaciens* in a recent study^45^, and other species clustered with *Treponema* reported from termites^44^. The roles of different *Treponema* species in the gut still need to be explored.

The presence of pathogenic *Leptospira* in great apes’ stool has never been reported. The high *Leptospira* spp. (pathogenic) prevalence observed and the identification of *L. interrogans* serovar Grippotyphosa in semi-captive bonobo feces was surprising (Fig. 4). It is important to note that Lola bonobo dormitories may be easily approachable for different species of wild and peridomestic rodents that can be a source of bonobo infection. In addition, bonobo feces could be contaminated by their own urine. NHPs might be sensitive to *Leptospira* infection, as an outbreak of severe leptospirosis was reported in capuchin (*Cebus*) monkeys^46^. *Leptospira* in the feces of wild bonobos could also be due to environmental contamination as samples were collected from the ground. Furthermore, the extent of *Leptospira* transmission between humans and NHPs is unknown.

*Strongyloides stercoralis* was found only in wild bonobos. Recent studies revealed the presence of *S. stercoralis* in human communities in contact with gorillas in the Congo^35^ and with long-tailed macaques in Thailand^47^.

African NHPs were reported to be reservoirs/hosts of *Oesophagostomum* roundworms^35,48^. Eight species of *Oesophagostomum* have been recognized so far to occur in NHPs^49^. Among them, *O. bifurcum, O. stephanostomum* and *O. aculeatum* are also reported in humans^50^. Central African gorillas and chimpanzees were reported to be infected (with sometimes fatal outcomes) by *O. stephanostomum* and, probably, by human-borne *O. bifurcum*^51^. The same two species seem to be responsible for endemic human esophagostomiasis in Ghana and Togo^52^. Subsequently, isolated cases have been described in Malaysia, Indonesia, Brunei, Brazil and several African countries (Ghana, Togo but also Zimbabwe, Ethiopia, Cote d’Ivoire, Uganda and Nigeria)^53^. We identify in our case *O. stephanostomum* in semicaptive and wild bonobos*,* and *O. bifurcum* in wild bonobos. The question concerning the role of great apes in the epidemiology of human nodular esophagostomosis remains open. Only the analysis of parasite population genetics can resolve the extent to which zoonotic transmission occurs.

Uncommonly, *T. solium* was identified in both bonobo populations. Cysticerci, presumably caused by *T. solium*, have been described in apes (gibbons and chimpanzees), New World monkeys (squirrel monkeys and marmosets), Old World monkeys (rhesus monkeys, baboons, mangabeys, patas monkeys, langurs, and vervets) and prosimians (lemurs)^54^.

*Trypanosoma theileri* was identified in Lola sanctuary bonobos. Usually, *T. theileri* infects *Bovinae* (cattle, buffalo, yaks, and some antelopes) and is prevalent in cattle throughout the world^55^. A previous study suggested that trypanosomiasis has been recorded among humans within the area of occurrence of bonobos and appears to be the most important disease shaping the area of occupancy of bonobos within their overall extent of occupancy^56^. Here, however, we also cannot exclude the possibility of contamination of bonobo feces by *Trypanosoma*-infected arthropods. Uncharacterized *Nematoda* and *Kinetoplastida* spp. are found in the two sites and need further exploration. The surveillance of parasitic infection in bonobos is of great importance for conservation and public health. Using the primate–parasite network, the role of different NHPs was evaluated for the probability of sharing parasitic infectious diseases with humans. Apes, as well as monkeys, such as baboons and macaques, were shown to be infected with many parasites identified as emerging infectious diseases in humans^57^.

One of the strengths of this study is the analysis of 91 stool samples from two different collection sites, Lola and Ekolo. By comparing the two collection sites, it was possible to establish a significant difference between Lola (animals in semicaptivity) and Ekolo (reintroduced into the wild), markedly in the case of *Giardia lamblia*, which was detected only in captive populations, as well as *Mycoplasma* and *Salmonella* spp. However, for the latter two pathogens, no significant difference between the two sites could be demonstrated possibly due to the number of samples from Ekolo being too low. It is, however, important to mention that there is possible degradation of the samples due to their collection in nature, and the prevalence of these microorganisms remains underestimated, even more so for extraintestinal microorganisms. *Treponema* spp. (non-*pallidum*) and *Mycobacterium* spp. (non-*tuberculosis*) were more significantly identified in Lola bonobos, suggesting possible transmission between humans and bonobos. Indeed, Lola is a sanctuary for the conservation and protection of orphaned bonobos. The animals cared for in the sanctuary are animals with an immature immune system, which makes them sensitive to possible exchange of bacterial flora with personnel. This characteristic should be considered when identifying bonobos as a potential reservoir of emerging infectious diseases. In addition, it has been shown that direct contact is not necessary to contaminate bonobos^58^. In Lola, bonobos are cared for by people who have to clean and prepare the housing areas. This maintenance work involves constant direct contact between them, which increases the risk of sharing pathogens and interspecies transmission.

Additionally, we looked for sexually transmitted pathogens in the current study (*Chlamydia* spp*.*, *T. pallidum*, *N. gonorrhea,* Simian HIV and papillomaviruses) because of sex-based conciliation practices in bonobos. All results were negative. The diversity of the nature of the samples would also broaden the range of pathogens not found in stool and provide a clearer diagnostic vision in bonobos. It would then be interesting in the future to use other types of excreta and biological fluids from these animals, at least for the populations in the sanctuary.

Two samples were of particular interest because they were positive for Astrovirus and other agents, including *Treponema* spp., *Mycobacterium* spp. and *O. stephanostomum*. One was also found to be positive for *Acinetobacter* spp. and another for *Giardia lamblia*. Both animals were from Lola. The fact that these pathogens were found in feces may suggest a latency period that allows pathogens to better adapt and/or induce pathogenicity in the host, the host tolerates infection and has an unrelated response^59,60^, or the pathogens simply are commensal and part of the animal’s intestinal microbiota.

For the first time, bacteria such as *Mycobacterium* spp*.*, *Salmonella* spp., *Acinetobacter* spp., and *Mycoplasma* spp. have been found in bonobo feces, as well as protozoa such as *T. theileri, B. saltans* and *G. lamblia* or *Astrovirus*. Finally, the results presented above can also be explained by the sample collection method, their transport and storage. Standardized collection conditions were maintained, and samples were then transported in alcohol from the DRC and maintained at − 80 °C. In addition, a number of missed organisms because of PCR failures due to primer mismatches is possible. Some microorganisms are highly diverse, especially in this part of the world (example: picornaviruses) and we can imagine primers design against a narrow set of these microorganisms would miss a lot of diversity. Consequently, it would be interesting to combine these results with a set of fresh stool samples collected from the same sites from which microorganisms were isolated.

## Methods

### Ethic statement, animals and study area

**Figure 8:**
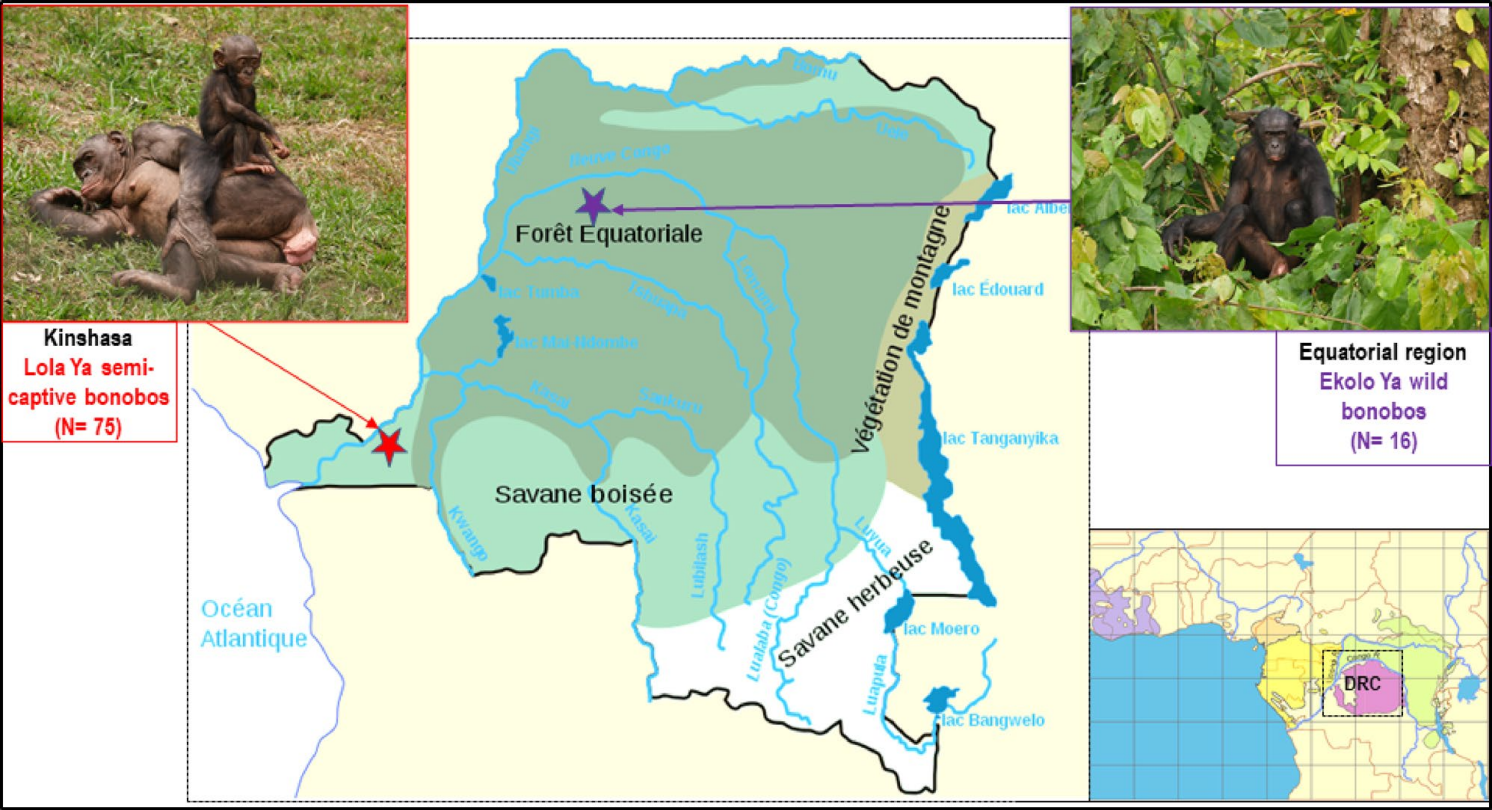
Study area. Samples from Lola Ya Bonobo (n = 75) where animals live in a sanctuary dedicated to the rehabilitation of orphan bonobos near Kinshasa, they are in permanent contact with humans. Samples from site Ekolo Ya bonobo (n = 16) were collected in nature where bonobos were introduced into the wild in the equatorial region. Figure modified from: https://docplayer.fr/162905495-Republique-democratique-du-congo-ministere-de-l-environnement-et-developpement-durable.html.

This study was based on 91 samples of bonobo feces collected in August 2017 from two collection sites in the Democratic Republic of Congo (DRC): 75 samples were collected in Lola Ya Bonobo (Lola), Kinshasa suburbs, a sanctuary for the protection, rehabilitation and reintroduction of orphaned bonobos; and 16 in Ekolo Ya Bonobo (Ekolo), Equateur region, a 20,000-hectare section of tropical forest dedicated to the reintroduction of bonobos (Fig. 8). The samples were aliquoted in alcohol and stored at − 80 °C upon arrival at the IHUMéditérannée Infection lab, Marseille.

The Public Health Ministry of the DRC has given its agreement for sample export (N° 482 INRB/DG of 01/09/17). Bouches-du-Rhône prefecture, in Marseille (France), has authorized the import of samples (N° 16/17 of 06/27/17). Finally, the feces of this species are not subject to an import–export permit for international circulation.

### DNA and RNA extraction

Nucleic acids (DNA/RNA) were extracted using the Qiagen Virus Mini Kit v2.0 (Qiagen, Courtaboeuf, France) for viral nucleic acid and the QIAamp DNA Mini Kit (Qiagen) for bacteria and parasites using an EZ1 biorobot (Qiagen). The DNA and RNA from each sample were extracted twice. This protocol included sample preparation with proteinase K, followed by mechanical stool lysis with tungsten beads (Qiagen, Courtaboeuf, France) using a FastPrep-24 5G Grinder. The supernatant was then recovered and incubated overnight at 56 °C. According to the manufacturer’s instructions, 130 µL of viral DNA/RNA and 200 µL of extracted DNA were collected in elution tubes, aliquoted in individual PCR tubes to an amount of 50 µL of pure extracted DNA/RNA, with another aliquot of 50 µL of DNA/RNA diluted to one-tenth, and finally a third aliquot of 50 µL of DNA/RNA diluted to one-hundredth. The original elution tubes containing the pure extractions were stored at − 20 °C for DNA and − 80 °C for RNA.

Bacterial DNA extraction was controlled by amplifying the 16S rRNA gene for all bacteria using a real-time PCR system (qPCR). Viral nucleic acid extraction was performed after adding 10 µL of internal controls in the extraction tubes, namely, Enterobacteria phage T4 (T4) and Enterobacteria phage MS2 for DNA and RNA controls, respectively. The extraction and dilutions were controlled by qPCR targeting the phages T4 and MS2 (Table S3).

### Reverse transcription (cDNA synthesis)

First-strand cDNA was synthesized using the MMLV-RT kit (Invitrogen, Carlsbad, CA, USA) according to the manufacturer’s protocol. The reaction mixture was prepared in a volume of 50 µL including 11 µL of MgCl_2_, 5 µL of Buffer 10×, 10 µL of dNTP (10 mM), 2.5 µL of hexameres at 1:10 dilution, 1.25 µL of RT-Multiscribe, 1 µL of RNAse, 9.25 µL of ultra-purified DNAse-RNAse-free water and finally 10 µL of DNA/RNA template extracted by EZ1. The RT-reaction was performed in a thermocycler (Applied Biosystem) with three thermal steps: 25 °C for 10 min, 48 °C for 30 min and 95 °C for 5 min followed by a pause step at 4 °C.

### Quantitative real-time PCR assays (qPCR)

The qPCR amplifications were performed in a CFX96 Real-Time system (Bio-Rad Laboratories, Foster City, CA, USA) after activating the readers of the dyes (FAM and/or VIC) used in each qPCR system. This method was used for the detection of parasites, viruses and bacteria of interest, using PCR systems for the detection of the pathogens studied here (Tables S1–3). The qPCR reactions were carried out in a final volume of 20 µL, containing 5 µL of DNA/cDNA template and 10 µL of Master Mix Roche (Eurogentec). The volume of each primer per reaction was 0.5 µL, with 0.5 µL of both UDG and each probe, and finally, the volume was brought to 20 µL using ultra-purified DNAse-RNAse-free water. The TaqMan cycling conditions included two hold steps at 50 °C for 2 min, followed by 95 °C for 15 min and 40 cycles of two steps each (95 °C for 30 s and 60 °C for 30 s). The PCR systems used for the study are detailed in (Table S4). Each PCR plate contains 96 wells; however, it was decided to run 50 samples per plate to avoid contamination. To confirm the results, samples that tested positive were retested using pure solutions and diluted to one-hundredth.

### Genetic amplification by standard PCR and sequencing

For gene amplifications, PCRs were performed in a total volume of 50 µL, consisting of 25 µL of AmpliTaq Gold master mix, 18 µL of ultra-purified water DNAse-RNAse free, 1 µL of each primer and 5 µL of DNA/cDNA template. The thermal cycling conditions were as follows: incubation step at 95 °C for 15 min, 40 cycles of 1 min at 95 °C, 30 s for the annealing at a different melting temperature for each PCR assay, 30 s to 1.5 min of elongation time at 72 °C (according to the fragment length), followed by a final extension for 5 min at 72 °C (Table S4). PCR amplification was performed in a Peltier PTC-200 model thermal cycler (MJ Research Inc., Watertown, MA, USA). The results of amplification were visualized by electrophoresis on a 2% agarose gel. The purification of PCR products was performed using NucleoFast 96-well PCR plates (Macherey Nagel EURL, Hoerdt, France) according to the manufacturer’s instructions. The amplicons were sequenced using the Big Dye Terminator Cycle Sequencing Kit (Perkin Elmer Applied Biosystems, Foster City, CA, USA) with an ABI automated sequencer (Applied Biosystems). The obtained electropherograms were assembled and edited using ChromasPro software (ChromasPro 1.7, Technelysium Pty Ltd., Tewantin, Australia) and compared with those available in the GenBank database by NCBI BLAST (https://blast.ncbi.nlm.nih.gov/Blast.cgi). Obtained sequences for each gene, for each pathogen, from positive samples were aligned with those available in the GenBank database for the same gene. Maximum-likelihood or the neighbor joining method was used to infer the phylogenetic analyses, and tree reconstruction was performed using MEGA software version 7 (https://www.megasoftware.net/)^61^. Bootstrap analyses were conducted using 1000 replicates.

For *Treponema* spp., since the 16S rRNA-based PCR^62^ did not allow identification, we developed a set of primers (F1, F2, R1, and R2) (Table S4) targeting the 23S rRNA of *Treponema* spp. First, sequences (Supplementary material S1) were aligned using BioEdit v 7.0.5.3 software^63^ to reveal conserved areas suitable as target regions for specific primers. This region was submitted to Primer3 software v. 0.4.0 (http://primer3.ut.ee/) to determine valuable candidate primers and probes, and selection was based on the criteria for primer and probe design. Degenerated nucleotides were used to achieve the maximum sensitivity within the genus *Treponema*. In the same manner, we developed sets of primers targeting the 16S, LipL 32, LipL 41, LipL 71, and Sec Y genes of *Leptospira* spp. (Table S4).

Settings for the PCR primers were in accordance with the guidelines as described by Apte and Daniel^64^ and as recommended by Invitrogen and Applied Biosystems. Melting temperatures, secondary structures and the possibility for primer-dimers were tested using the free online software Oligo Analyzer 3.1^65^. All primer sequences were also checked for their specificity in an NCBI BLAST nucleotide sequence similarity search^66^. Furthermore, they were checked within the DNA databases of metazoans (taxid:33208), vertebrates (taxid:7742), bacteria (taxid:2), arthropods (taxid:6656), primates (taxid:9443), *Canidae* (taxid:9608), *Felidae* (taxid:9682) and humans (taxid:9605) as previously described^67^. Primers were synthesized by Eurogentec (Liège, Belgium).

### Microorganisms screened

To optimize time and resources, zoonotic pathogens and, in particular, those routinely researched at the IHU Marseille lab, were studied (Table 3).

### Statistical analyses

To determine if there is a difference in the frequency of microorganisms between the bonobos living in semicaptivity (Lola) and the wild (Ekolo), a Z test was performed. Significant differences were considered at p < 0.05.

## Supplementary Information

Supplementary Information.

## Data Availability

All data are included in the manuscript. The newly generated sequences were deposited in the GenBank database under the accession numbers: MW046203-MW046205 (*3D pol*) of EMCV; MW067650-MW067654 (*LipL41*) of *Leptospira* spp.; MT257118-MT257134 (*23S*) of *Treponema* spp.; MW040123-MW040123 (ITS2) of *Oesophagostumum* spp.; MT890583 (*18S*) of *Oesophagostumum* spp.; MT886281-MT886283 (*18S*) of *Trypanosma theileri;* MT886284 (*18S*) of *Bodo saltans*.
