# Author Correction: Potential zoonotic pathogens hosted by endangered bonobos

**DOI:** 10.1038/s41598-021-92698-8

**Published:** 2021-06-17

**Authors:** Hacène Medkour, Sergei Castaneda, Inestin Amona, Florence Fenollar, Claudine André, Raphaël Belais, Paulin Mungongo, Jean‑Jacques Muyembé‑Tamfum, Anthony Levasseur, Didier Raoult, Bernard Davoust, Oleg Mediannikov

**Affiliations:** 1 Aix Marseille Univ, IRD, AP-HM, MEPHI, IHU-Méditerranée Infection, Marseille, France; 2 IHU-Méditerranée Infection, Marseille, France; 3 Aix Marseille Univ, IRD, AP-HM, SSA, VITROME, IHU-Méditerranée Infection, Marseille, France; 4 Les Amis Des Bonobos du Congo, Kinshasa, Democratic Republic of the Congo; 5 National Institute of Biomedical Research (INRB), Kinshasa, Democratic Republic of the Congo

Correction to: *Scientific Reports* 10.1038/s41598-021-85849-4, published online 18 March 2021

The original version of this Article contained an error in the Discussion section, where,

“It seems that the sanctuary has not followed the international recommendations for reintroduction^38^.”

now reads:

“In our study, the Lola Ya sanctuary followed the international recommendations for reintroduction^38^.”

The original Article has been corrected.

