# Supplementary material for: Potential zoonotic pathogens hosted by endangered bonobos
Source: Sci Rep. 2021 Mar 18;11:6331. doi: 10.1038/s41598-021-85849-4 (PMC7973442; doi:10.1038/s41598-021-85849-4)
Supplement: Supplementary file 1 — Supplementary Information. [file 41598_2021_85849_MOESM1_ESM.pdf]

Potential Zoonotic Pathogens Hosted by Endangered Bonobos

Hacène Medkour [1,2], Sergei Castaneda[1,2], Inestin Amona[2,3], Florence Fenollar[2,3], Claudine André[4], Raphaël Belais[4], Paulin Mungongo[4], Jean-Jacques Muyembé-Tamfum[5], Anthony Levasseur[2,3], Didier Raoult[1,2], Bernard Davoust[1,2], Oleg Mediannikov[1,2*]

[1]Aix Marseille Univ, IRD, AP-HM, MEPHI, IHU-Méditerranée Infection, Marseille, France

[2]IHU-Méditerranée Infection, Marseille, France

[3]Aix Marseille Univ, IRD, AP-HM, SSA, VITROME, IHU-Méditerranée Infection, Marseille, France

[4] Les Amis des Bonobos du Congo, Kinshasa, Democratic Republic of the Congo.

[5] National Institute of Biomedical Research (INRB), Kinshasa, Democratic Republic of the Congo.

***Correspondence:**
Oleg Mediannikov; Aix Marseille Univ, IRD, AP-HM, MEPHI, IHU-Méditerranée Infection, Marseille, France

mail: olegusss1@gmail.com

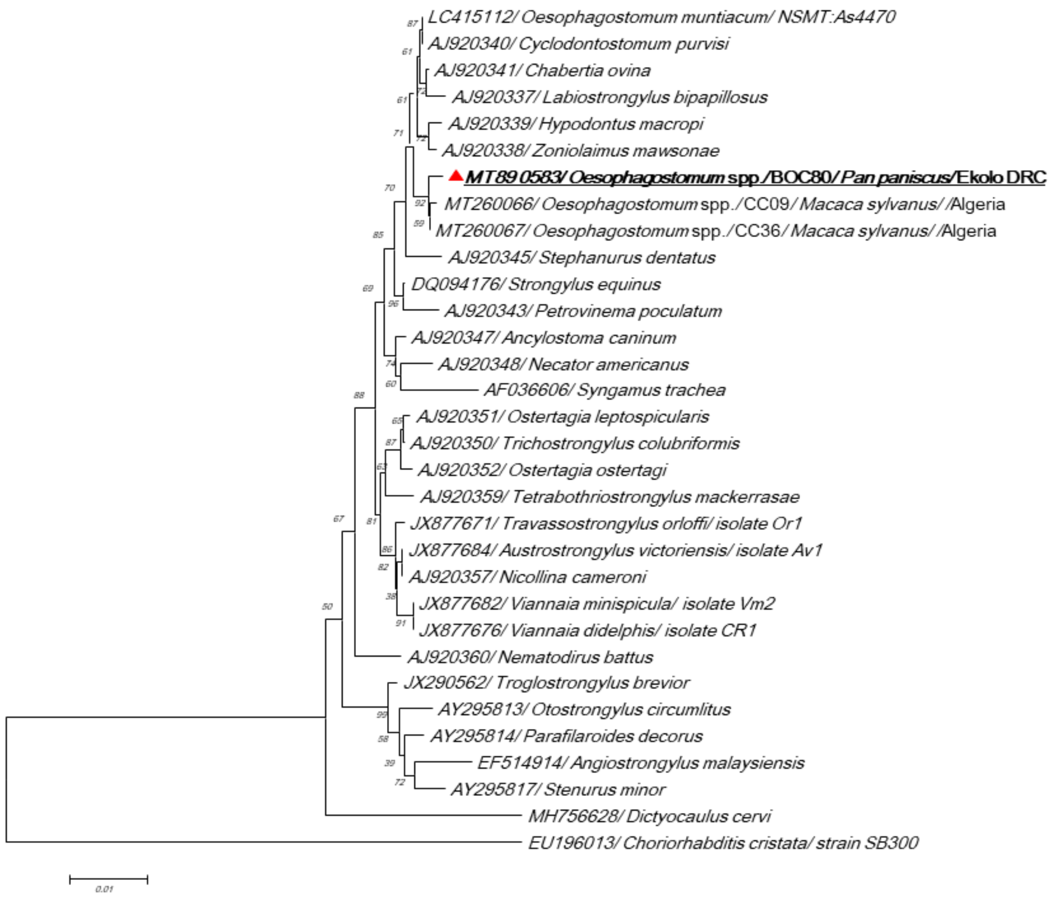

Fig. S1 Phylogenetic tree for *Nematoda* sp. detected on bonobo samples. The evolutionary history based on the *18S rRNA* partial gene, was inferred using the Neighbor-Joining method. Sequences are identified as follows: accession number/species/ strain/ host/ country. Sequences identified in this study showed >99.2 identity with *Oesophagostomum* spp.; sequences almost similar were also detected on Barbary macaques from Algeria. There were a total of 1083 positions in the final dataset. Evolutionary analyses were conducted in MEGA7.

Table S1. Primer and probe sequences used in this study for the bacteria

Bacteria	Target gene	Primer name	Sequence (5'- 3')	Source
Acinetobacter spp.	rpoB	Acineto-rpoBF	TACTCATATACCGAAAAGAAACGG	Bouvresse et al. 2011
		Acineto-rpoBR	GGYTTACCAAGRCTATACTCAAC	
		AcinetorpoB	6-FAM-CGCGAAGATATCGGTCTSCAAGC-TAMRA	
A. baumannii	*OmpA/MotB*	R	CGCTCTTGCCAGCATAAAGA	Ly et al. 2019
		F	TCAACATCACAATCTTTAGTAGCTGA	
		P	FAM_AAGTCGCCAAGAAACCTTGA-TAMRA	
Anaplasmataceae	23S	TtAna_F	TGACAGCGTACCTTTTGCAT	Dahmani et al. 2015
		TtAna_R	GTAACAGGTTCGGTCCTCCA	
		TtAna_P	6FAM- GGATTAGACCCGAAACCAAG-TAMRA	
Wolbachia spp.	16S	all.Wol.16S.301-F	TGGAACTGAGATACGGTCCAG	Laidoudi et al. 2020
		all.Wol.16S.301-R	GCACGGAGTTAGCCAGGACT	
		all.Wol.16S.301-P	6FAM-AATATTGGACAATGGGCGAA-TAMRA	
Bartonella spp.	ITS	Barto_ITS3_F	GATGCCGGGGAAGGTTTTC	Mourembou et al. 2015
		Barto_ITS3_R	GCCTGGGAGGACTTGAACCT	
		Barto_ITS3_P	6FAM- GCGCGCGCTTGATAAGCGTG-TAMRA	
Borrelia spp.	16S	Bor16S3F	AGC CTT TAA AGC TTC GCT TGT AG	Parola et al. 2011
		Bor16S3R	GCC TCC CGT AGG AGT CTG G	
		Bor16S3P	6FAM-CCG GCC TGA GAG GGT GAA CGG-TAMRA	
Coxiella burnetii	IS1111A	CB_IS1111_0706F	CAAGAAACGTATCGCTGTGGC	Mediannikov et al. 2010
		CB_IS1111_0706R	CACAGAGCCACCGTATGAATC	
		CB_IS1111_0706P	6FAM- CCGAGTTCGAAACAATGAGGGCTG-TAMRA	
Chlamydia spp.	23S	CHL23SUP	GGGGTTGTAGGGTYGAGRAIAWRRGATC	DeGraves et al. 2003
		CHL23SDN	GAGAGTGGTCTCCCCAGATTCARACTA	
		CHL23LCR	6FAM- CCTGAGTAGRRCTAGACACGTGAAAC-TAMRA	
Atopobium vaginae	16S	Atop_F	CCCTATCCGCTCCTGATACC	Fenollar et al. 2008
		Atop_R	CCAAATATCTGCGCATTTCA	
		Atop_P	VIC-GCAGGCTTGAGTCTGGTAGGGGA-TAMRA	
Neisseria gonorrhoeae	Hypothetical proteine	Ngono_2F	CATCAGCGCAATCCCTATGA	Hopkins et al. 2010
		Ngono_2R	TGGTTGTCCTCAACCGTGTC	
		Ngono_2 P	6FAM- CCACGGCGCTTCCTGATGGG-TAMRA	
	PorA	Pap-Tm-F	CAGCATTCAATTTGTTCCGAGTC	
		Pap-Tm-R	GAACTGGTTTCATCTGATTACTTTCCA	
		Pap-Tm-P	6FAM- CGCCTATACGCCTGCTACTTTCACGC	
Gardnerella vaginalis	Cpn60	Gvaginalis_F	CGCATCTGCTAAGGATGTTG	Fenollar et al. 2008
		Gvaginalis_R	CAGCAATCTTTTCGCCAACT	
		Gvaginalis_P	VIC-TGCAACTATTTCTGCAGCAGATCCT-TAMRA	
Helicobacter pylori	23S rRNA	HPY_F	AGGTTAAGAGGATGCGTCAGTC	Menard et al. 2002
		HPY_R	CGCATGATATTCCCATTAGCAGT	
		HPY_P	6FAM-TGGGAGCTGTCTCAACCAGAGATTCAGTG-TAMRA	
Leptospira spp.	16S	Lepto 171F	CCCGCGTCCGATTAG	Smythe et al. 2002
		Lepto 258R	TCCATTGTGGCCGRA/GACAC	
		Lepto P	6-FAM- CTCACCAAGGCGACGATCGGTAGC-TAMRA	
Mycoplasma spp.	ITS1	Mycop_ITS1_F	GGGAGCTGGTAATACCCAAAGT	Cohen et al. 2011
		Mycop_ITS1_R	CCATCCCCACGTTCTCGTAG	
		Mycop_ITS1_P	6FAM- GCCTAAGGTAGGACTGGTGACTGGGG-TAMRA	
Mycobacterium spp.	ITS	Mycob_ITS_F	GGGTGGGGTGTGGTGTTTGA	Bruijnesteijn et al. 2004
		Mycob_ITS_R	CAAGGCATCCACCATGCGC	
		Mycob_ITS_P	6FAM- TGGATAGTGGTTGCGAGCATC-TAMRA	

	ITS	Mtub_ITS_P	6FAM- GCTAGCCGGCAGCGTATCCAT-TAMRA	
M. genitalium	*FusA*	Mgen_fusA_F	CTGGGAAAACCACCACATCA	Edouard et al. 2017
		Mgen_fusA_R	CACTGAAGTGGCTGCAGAGG	
		Mgen_fusA_P	6FAM-TGGTGAATCAGTGATGGACTGGATGGA-TAMRA	
Rickettsia felis	*bioB*	Rfelis_0527_F	ATGTTCGGGCTTCCGGTATG	Mourembou et al. 2015
		Rfelis_0527_R	CCGATTCAGCAGGTTCTTCAA	
		Rfelis_0527_P	6FAM- GCTGCGGCGGTATTTTAGGAATGGG-TAMRA	
Salmonella spp.	*invA*	invA_F	TCTGTTTACCGGGCATACCA	Mourembou et al. 2015
		invA_R	CACCGTGGTCCAGTTTATCG	
		invA_P	6FAM- CCAGAGAAAATCGGGCCGCG-TAMRA	
	sipC	sipC_F	GTCAGGCGTCGTAAAAGCTG	Mourembou et al. 2015
		sipC_R	ACGTCGACTGGTGGTACTGG	
		sipC P	6FAM- CTCCAGGCGCGAACAGCTGG-TAMRA	
Salmonella typhi	*hypothetical protein*	Styphi_put_F	TCTCATGCTGCGACCTCAAA	
		Styphi_put_R	TTCATCCTGGTCCGGTGTCT	
		Styphi_put_P	6FAM- GCTTTTTGTGAAGCAACGCTGGCA-TAMRA	
Salmonella typhi/paratyphi	*narG*	Styphi_narG_F	GCGCCACATCTTCATCAGAC	
		Styphi_narG_R	CCCGTCCTGATATGCCAAAC	
		Styphi_narG_P	6FAM- AGTAACTTGCCCCGCGCGGG-TAMRA	
Staphylococcus aureus	*NucA*	Saur_NucA_F2	GTTGTGGATGGTGATACATTTATTGC	Mourembou et al. 2015
		Saur_NucA_R2	CCAAATGGTTGTACAGGCGTATTC	
		Saur_NucA_P2	6FAM- AGGCTTATAGGGGTTGATACGCCAGAAACGG-TAMRA	
	Amidohydrolase	Saur_Amido_F	CCTCGACAGGTAACGCATCA	
		Saur_Amido_R	AAACTCCTATCGGCCGCAAT	
		Saur_Amido_P	6FAM- TGCAATGGTAGGTCCTGTGCCCA	
Treponema spp.	23S	forw2570	CGCCGTCACTTAACGGAYA	This study
		rev2660	GATGCGATGAGCCGACAT	
		sonde2620	6FAM- CCCCAAGAGTTCACATCGAC-TAMRA	
Treponema pallidum	PolA	polA SyphT_F1	GTCGAGACTGAAAAGGAGTGCA	Leisli et al. 2007
		SyphT_R1	GTGAGCGTCTCATCATTCCAAAG	
		SyphT_P1	FAM- TGCTGTGCAGGATCCGGCATATGTCC-TAMRA	
	Fla A	Tpal_flaA_F	GCGGTTGCACAGTGGGAG	Salazar et al. 2007
		Tpal_flaA_R	CAGCATGGGCGACAGGAT	
		Tpal_flaA_P	FAM- TTGTGCTGAATTCTTCCGCGCG-TAMRA	
Tropheryma whipplei	WiSP family protein (whi 2)	T_whi2_F	TTGTGTATTTGGTATTAGATGAAACAG	Fenollar et al. 2010
		T_whi2_R	CCCTACAATATGAAACAGCCTTTG	
		T_whi2_P	6FAM- GGGATAGAGCAGGAGGTGTCTGTCTGG-TAMRA	
	WiSP family protein (whi 3)	T_whi3_F	TTGTGTATTTGGTATTAGATGAAACAG	
		T_whi3_R	CCCTACAATATGAAACAGCCTTTG	
		T_whi3_P	6FAM-GGGATAGAGCAGGAGGTGTCTGTCTGG-TAMRA	
Listeria monocytogenes	hlyQ	Lmo_hlyQ_F	CATGGCACCACCAGCATC	Fal et al. 2019
		Lmo_hlyQ_R	ATCCGCGTGTTTCTTTTCGA	
		Lmo_hlyQ_P	6FAM- CGCCTGCAAGTCCTAAGACGCCA-TAMRA	
Clostridium difficile	C. difficile Toxine A	Cdiff_tcdA_F	GGTAATAATTCAAAAGCGGCT	Luna et al. 2011
		Cdiff_tcdA_R	AGCATCCGTATTAGCAGGTG	
		Cdiff_tcdA_P	6FAM- AGCCTAATACAGCTATGGGTGCGAAG-TAMRA	
	C. difficile Toxine B	Cdiff_tcdB_F	GAAAGTCCAAGTTTACGCTCAAT	
		Cdiff_tcdB_R	GCTGCACCTAAACTTACACCA	
		Cdiff_tcdB_P	VIC- ACAGATGCAGCCAAAGTTGTTGAATT-TAMRA	
Yersinia pestis	pLA	YPpla S	GTAATAGGTTATAACCAGCGCTT	

		YPpla R	AGACTTTGGCATTAGGTGTG	Riehm et al. 2011
		YP pla TM	6FAM-ATGCCATATATTGGACTTGCAGGCCAGT-BBQ	
Vibrio cholerae	toxR	toxR-F1	CCGAATAACCACCCTGATCTTT	Greig et al. 2018
		toxR-R1	ACCTGTGGCAATGACTTCTATC	
		toxR-Pr	6FAM -ACTGGCTACCGTCAATCGAACTGT- TAMRA	

Table S2. Primer and probe sequences used in this study for the parasites

**Parasites**	**Target gene**	**Primer name**	**Sequence (5'- 3')**	**Source**
Ancylostoma duodenale	ITS2	Ad125F	GAATGACAGCAAACTCGTTGTTG	Verweij et al. 2007
		Ad195R	ATACTAGCCACTGCCGAAACGT	
		Ad155-XS	6FAM-ATCGTTTACCGACTTTAG-MGB	
Ascaris lumbricoides	ITS1	Alum96F	GTAATAGCAGTCGGCGGTTTCTT	Wiria et al. 2010
		Alum183R	GCCCAACATGCCACCTATTC	
		Alum124T	6FAM- TTGGCGGACAATTGCATGCGAT-TAMRA	
Cryptosporidium parvum; C. hominis	hsp70 gene	1PSF	AACTTTAGCTCCAGTTGAGAAAGTACTC	Garcés-Sanchez et al. 2002
		1PSR	AACTTTAGCTCCAGTTGAGAAAGTACTC	
		Crypt P	6FAM-AATACGTGTAGAACCACCAACCAATACAACATC-TAMRA	
Cyclospora cayetanensis	18s	Cyclo250F	TAGTAACCGAACGGATCGCATT	Verweij et al. 2003
		Cyclo350R	AATGCCACGTAGGCCAATA	
		Cyclo281T	6FAM-CCGGCGATAGATCATTCAAGTTTCTGACC-TAMRA	
Entamoeba histolytica	18S	Ehf	AACAGTAATAGTTTCTTTGGTTAGTAAAA	Roy et al. 2005
		Ehr	CTTAGAATGTCATTTCTCAATTCAT	
		Ehp	6FAM-ATTAGTACAAAATGGCCAATTCATTCA-TAMRA	
Enterobius vermicularis	5S	EnterF	TTTCCAAGCCACAGACTCAC	Sow et al. 2017
		EnterR	ATTGCTCGTTTGCCGATTAT	
		EnterP	TCATGTCTGAGCCGGAACGAGA	
Filariae	28S	qFil-28S-F	TTGTTTGAGATTGCAGCCCA	Laidoudi et al. 2019
		qFil-28S-R	GTTTCCATCTCAGCGGTTTC	
		qFil-28S-S	6FAM-CAAGTACCGTGAGGGAAAGT-TAMRA	
Giardia lamblia (intestinalis or duodenalis)	18S	Giardia-80F	GACGGCTCAGGACAACGGTT	Verweij 2004
		Giardia-127R	TTGCCAGCGGTGTCCG	
		Giardia-105T	6FAM-CCCGCGGCGGTCCCTGCTAG-TAMRA	
Kinetoplastida spp.	28S	F. 24a.5198	AGTATTGAGCCAAAGAAGG	Medkour et al. 2020
		R. 24a.5412	TTGTCACGACTTCAGGTTCTAT	
		P. 24a.5345	6FAM-TAGGAAGACCGATAGCGAACAAGTAG-TAMRA	
Leishmania spp.	18S	F	GGTTTAGTGCGTCCGGTG	Medkour et al. 2020
		R	ACGCCCCAGTACGTTCTCC	
		P	6FAM- CGGCCGTAACGCCTTTTCAACTCA-TAMRA	
Loa loa	LL20 15kDa ladder antigen	15r3-5	CGAAAAATTATAGGGGGAAAC	Touré et al. 1997
		15r3-6	TCGTAGACCAAACTGCGAAC	
		15r3-P	6FAM- TCAAGAGCCGATATACTGAAAGCTATC-TAMRA	
Mansonella spp.	ITS1	Forward	CCTGCGGAAGGATCATTAAC	Bassene et al. 2015
		Reverse	ATCGACGGTTTAGGCGATAA	
		Probe	6FAM-CGGTGATATTCGTTGGTGTCT-TAMRA	
Mansonella perstans	ITS1	Forward	AGGATCATTAACGAGCTTCC	
		Reverse	CGAATATCACCGTTAATTCAGT	
		Probe	6FAM-TTCACTTTTATTTAGCAACATGCA-TAMRA	
Necator americanus	ITS2	Na58F	CTGTTTGTCGAACGGTACTTGC	Verweij 2007
		Na158R	ATAACAGCGTGCACATGTTGC	
		Na81T	6FAM- CTGTACTACGCATTGTATAC -MGB	
Nematoda	5S	qNem.5S.1f	ACCACGTTGAAAGCACGMC	

		qNem.5S.110r	TGTCTACAACACCTSGRATTCC	Laidoudi et al. 2020
		qNem.5S.38p	6FAM-AGTTAAGCAACGTTGGGCC-TAMRA	
Plasmodium spp.	Cox	Plasmo_cox_15_F	AGGAACTCGACTGGCCTACA	Mourembou et al. 2015
		Plasmo_cox_16_R	CCAGCGACAGCGGTTATACT	
		Plasmo-cox_P	6FAM- CGAACGCTTTTAACGCCTGACATGG-TAMRA	
Strongyloides stercoralis	18S	Stro-1530F	GAATTCCAAGTAAACGTAAGTCATTAGC	Verweij et al. 2009
		Stro-1630R	TGCCTCTGGATATTGCTCAGTTC	
		Stro-1586T	6FAM-ACACACCGGCCGTCGCTGC-TAMRA	
Physaloptera spp.	12S	Phy.12S.f.204	GAATTGGATTAGTACCCAAGTAAGTG	Laidoudi et al. 2020
		Phy.12S.r.305	TGTTCCAAAAATCTTTCTAAGATCAG	
		Phy.12S.242p	VIC-GCGGGAGTAAAGTTAAGTTTAAACC-TAMRA	
Taenia saginata	ITS	Tsol_ 145F	ATGGATCAATCTGGGTGGAGTT	Praet et al. 2013
		Tsol_ 230R	ATCGCAGGGTAAGAAAAGAAGGT	
		Tsol_169Tq	6FAM-TGGTACTGCTGTGGCGGCGG-TAMRA	
Taenia solium	ITS	Tsag_F529	GCGTCGTCTTTGCGTTACAC	
		Tsag_R607	TGACACAACCGCGCTCTG	
		Tsag_581Tq	6FAM-CCACAGCACCAGCGACAGCAGCAA-TAMRA	
Toxoplasma gondii		Tgon_ITS1_F	GATTTGCATTCAAGAAGCGTGATAGTA	Jauregui et al. 2001
		Tgon_ITS1_R	AGTTTAGGAAGCAATCTGAAAGCACATC	
		Tgon_ITS1_P	6FAM-CTGCGCTGCTTCCAATATTGG-TAMRA	
Trichuris trichiura	18S	TrichF	TTGAAACGACTTGCTCATCAACTT	Liu 2013
		TrichR	CTGATTCTCCGTTAACCGTTGTC	
		TrichP	6FAM-CGATGGTACGCTACGTGCTTACCATGG-TAMRA	

Table S3. Primer and probe sequences used in this study for viruses

Virus	Target gene	Primer name	Sequence (5'- 3')	Source
DNA Extraction control	Phage T4	T4F	CCATCCATAGAGAAAATATCAGAACGA	Ninove et al. 2011
		T4R	TAAATAATTCCTCTTTTCCCAGCG	
		T4P	VIC- AACCAGTAATTTCATCTGCTTCTGATGTGAGGC-TAMRA	
RNA Extraction control	Phage MS2	MS2F	CTCTGAGAGCGGCTCTATTGGT	
		MS2R	GTTCCCTACAACGAGCCTAAATTC	
		MS2P	VIC- TCAGACACGCGGTCCGCTATAACGA-TAMRA	
Adenovirus (Adv.)	hexon gene	AQ1	GCCACGGTGGGGTTTCTAAACTT	Heim et al. 2003
		AQ2	GCCCCAGTGGTCTTACATGCACATC	
		AP	6FAM-TGCACCAGACCCGGGCTCAGGTACTCCGA-TAMRA	
Coronavirus	The ARGENE kit of the HCoH / HPIV r-gene complex, which allows the detection of 4 species of HCoH (NL63, 0C43, HKLU1 and 229E) and of HPIV (HPIV-1, HPIV2, HPIV3 and HPIV4), was used according to the manufacturer's recommendations (ARGENE, HCoH / HPIV r-gene, Biomerieux SA, France). (bioMérieux, https://www.biomerieux-diagnostics.com)			
Sarbecovirus	ORF1b-nsp14	HKU-ORF1b-nsp14F	TGGGGYTTTACRGGTAACCT	WHO, Korea
		HKU- ORF1b-nsp14R	AACRCGCTTAACAAAGCACTC	
		HKU-ORF1b-nsp141P	6FAM-TAGTTGTGATGCWATCATGACTAG-TAMRA	
	N	HKU-NF	TAATCAGACAAGGAACTGATTA	
		HKU-NR	CGAAGGTGTGACTTCCATG	
		HKU-NP	6FAM-GCAAATTGTGCAATTTGCGG- TAMRA	
Sars-Cov 2	E	E_Sarbeco_F1	ACAGGTACGTTAATAGTTAATAGCGT	Corman et al. 2020
		E_Sarbeco_R2	ATATTGCAGCAGTACGCACACA	
		E_Sarbeco_P1	FAM-ACACTAGCCATCCTTACTGCGCTTCG-QSY	
Enterovirus (EV)	5' UTR	HevFwd356	GCTGCGYTGGCGGCC	Tapparel et al. 2009
		EV PICO R	GAAACACGGACACCCAAAGTAGT	

		EV PICOPro	6FAM -CTCCGGCCCCTGAATGYGGCTAA-TAMRA	
Hepatitis A virus (HAV)	VP1	HavMrsRTfwd	GGTAGGCTACGGGTGAAAC	Motte et al. 2009
		HavMrsRTRev	GCGGATATTGGTGAGTTGTT	
		HavMrsPr	6FAM-CTTARGCTARTACTTCTATGAAGAGATGC-TAMRA	
Hepatitis E virus (HEV)	ORF3	HEVORF3-S	GGT GGT TTC TGG GGT GAC	Izopet et al. 2012
		HEVORF3-AS	AGG GGT TGG TTG GAT GAA	
		HEVORF3-P	6FAM -TGATTCTCAGCCCTTCGC- TAMRA	
Herpes simplex virus (HSV)	DNA polymerase gene	HSV DNA POL sens	CATCACCGACCCGGAGAGGGAC	Kessler et al. 2000
		HSV DNA POLrev	GGGCCAGGCGCTTGTTGGTGTA	
		HSV DNA POL PROBE	6FAM-CCGCCGAACTGAGCAGACACCCGCGC-TAMRA	
Norovirus GG2	ORF1 and ORF2	NV117	TCGACGCCATCTTCATTCA	Höhne et Schreier, 2004
		NV107a	AGCCAATGTTCAGATGGATG	
		NoroGGII_TM3	6FAM-TGGGAGGGCGATCGCAATCTGGC-TAMRA	
Parechovirus (HPeV)	Polyprotein gene	PeV_F	CTGGGGCCAAAAGCCA	Benschop et al. 2010
		PeV_R	GGTACCTTCTGGGCATCCTTC	
		PeV_P	6FAM- AAACACTAGTTGTAWGGCCC-TAMRA	
Rotavirus	NSP3	Rota-2-F	ACCATCTWCACRTRACCCTCTATGAG	Zeng et al. 2008
		Rota-2-R	GGTCACATAACGCCCCTATAGC	
		Rota-2-P	6FAM -AGTTAAAAGCTAACACTGTCAAA-TAMRA	
Simian immunodeficiency virus (SIV)	gag	SIVsmE660 Forward	GCAGAGACATCTAGTGGTGGAAAC	Monjure et al. 2014
		SIVsmE660 Reverse	GTAATTTCCTCCTCTGCCACTAGG	
		SIVsmE660 Probe	6FAM-AATGCCAGCAACAAGCAGACCAACAGCA IA-BKFQ	
Poxvirus	Humagglitunin	OPV_HA_F	TGATGCAACTCTATCATGTARTCG	Andreani et al. 2019
		OPV_HA_R	CAAGACGTCGCTTTTRGCAG	
		OPV_HA_P	6FAM- TGCTTGGTATAAGGAGCCCAATTCCA	
Sapovirus	Polymerase/ capsid junction	sapo.fwdA	ACC AGG CTC TCG CCA CCT A	Logan et al. 2007
		sapo.fwdB	ATT TGG CCC TCG CCA CCT A	
		sapo.rev	GCC CTC CAT YTC AAA CAC TAW TTT	
		sapo.probeA	6FAM -CTG TAC CAC CTA TGA ACC A-TAMRA	
		sapo.probeB	6FAM -TTG TAC CAC CTA TGA ACC A-TAMRA	
		sapo.probeC	6FAM -TGT ACC ACC TAT AAA CCA-TAMRA	
		sapo.probeD	6FAM -TGC ACC ACC TAT GAA C-TAMRA	

Table S4. Primers used in standard PCRs and sequencing.

**Microorganisms**	**Target gene**	**Primer name**	**Sequence (5'- 3')**	**Length and Tm**	**Source**
Papillomavirus (HPV)	L1 gene	MY09	GCMCAGGGWCATAATAATGG	2 runs of PCR, the first at Tm: 52°C; the second at Tm: 55°C.	Tamalet C et al. 2010
		MY11	CGTCCMARRGGAWACTGATC		
EMCV	3D Protein	P1	CCCTACCTCACGGAATGGGGCAAAG	Nested PCR: P1/F1 (400 bp) at 55°C; then F2/R2 (250 bp) at 55°C	This study
		F1	TTATWATTAGGGCIGGYTTG		
		F2	CTAGCAAAGACAGGRTAYAA		
		R2	ACGRAAIGGGGCAAAGAG		
ECMV64SPU/03 (Congo)		ECMV64SPU/03 F	CGCAAAGACAGGATATAAGATAACAC		
		ECMV64SPU/03 R	ACTCTTGCTTGCCAGAATGTA		
Adenoviruses (AdVs)	DNA polymerase	Fw Outer	TGATGCGYTTCTTACCTYTGGTYTCCATGAG	Nested PCR, ≈1400 bp at 58°C ≈250 bp at 55°C	Roy et al. 2009
		Rw Outer	AGTTYTACATGCTGGGCTCTTACCG		
		Fw inner	GTGACAAAGAGGCTGTCCGTGTCCCCGTA		

		Rw inner	TCACGTGGCCTACACTTACAAGCCAATCAC		
Metazoans	Cox1	dgLCO-1490	GGTCAACAAATCATAAAGAYATYGG	708 bp, Tm: 44°C	Folmer et al. 1994
		dgHCO-2198	TAAACTTCAGGGTGACCAAARAAYCA		
Nematoda	18S	Fwd.18S.631	TCGTCATTGCTGCGGTTAAA	1127–1155 bp at 54°C	Laidoudi et al. 2019
		Rwd.18S.1825r	GGTTCAAGCCACTGCGATTAA		
Helminth	28S	Hspec.28S. 5748f	GGTAAGGGAAGTCGGCAAAT	400 bp at 56°C	Medkour et al. 2020
		Hspec.28S.6394r	AGGGACAGTGGGAATCTCG		
Oesophagostomum spp.	ITS2	NC1	TTAGTTTCTTTTCCTCCGCT	1) NC1/ NC2 (280 - 400 bp), Tm: 50°C 2) NC2/ OesophITS2-21 (260 bp), Tm: 55°C	Ghai et al. 2014
		NC2	ACGTCTGGTTCAGGGTTGTT		
		OesophITS2-21	TGTRACACTGTTTGTCGAAC		
Kinetoplastida	18S	F720	GTTAAAGGGTTCGTAGTTGAA	750 bp, Tm: 50°C	Keita et al. 2020
		R1495	GACTACAATGGTCTCTAATCA		
Leptospira spp.	Adk	F	GGGCTGGAAAAGGTACACAA	531 bp, Tm: 53°C	Ahmed et al. 2006
		R	ACGCAAGCTCCTTTTGAATC		
	icdA	F	GGGACGAGATGACCAGGAT	674 bp, Tm: 53°C	
		R	TTTTTTGAGATCCGCAGCTTT		
	LipL41	F	TAGGAAATTGCGCAGCTACA	520 bp, Tm: 53°C	
		R	GCATCGAGAGGAATTAACATCA		
	rrs2	F	CATGCAAGTCAAGCGGAGTA	541 bp, Tm: 53°C	
		R	AGTTGAGCCCGCAGTTTTC		
	SecY	F	ATGCCGATCATTTTTGCTTC	549 bp, Tm: 53°C	
		R	CCGTCCCTTAATTTTAGACTTCTTC		
	LipL32	F	ATCTCCGTTGCACTCTTTGC	474 bp, Tm: 53°C	
		R	ACCATCATCATCATCGTCCA		
	16S	F 16S 37-56	CGGCGCGTCTTAAACATGCA	1125 pb, Tm: 53°C	This study
		F2 16S 397-419	GAAGGTCTTCGGATTGTAAAGT		
		R 16S 857-877	CACTCTTGCGAGCATAGTCC		
		R2 16S 1162-1183	ACATAAAGGCCATGAGGATTTG		
	23S	F 23S 1326-1345	CGGCGCGTCTTAAACATGCA	1100 bp, Tm: 53°C	
		F2 23S 1745-1764	TAAGGAACTCGGCAAATTAC		
		R 23S 1942-1962	AGCTTCGCATTGCTGCTAAC		
		R2 23S 2409-2434	GTCTCACAGTTAAGCTCCCTTATGCC		
	LipL32	F LipL32 34-53	GCACTCTTTGCAAGCATTAC	620 bp, Tm: 53°C	
		R LipL 32 653-673	ATCCTTTCACTTCACCTGGTT		
	LipL 41	F LipL41 83-103	ATCCGGTATTCCCGAAAGATA	600 bp, Tm: 50°C	
		R LipL41 682-700	CTTCRTCCAAWCCTTGTCC		
	LipL 71	F LipL71 437-456	CGAACCACATAACGTTTCCA	645 bp, Tm: 45°C	
		R LipL71 1073-1092	AAACAACAAYTAATCGATTC		
	Yidc	F Yidc 49-68	GGAATTTGGATGGGAGTGAA	880 bp, Tm: 51°C	
		F2 Yidc 348-363	TTCAAGTTGCTCGTAA		
		R Yidc 920-942	ATAAAATAACGAGAACCMGTTCC		
Treponema spp.	23S	F1- 3274	GGGAGTGAGACTGCGIGCG	F1/R2: ≈900 bp, Tm: 57°C	This study
		F2- 3592	GAGAATGCAGGYATAAGTA		
		R1- 3671	CCGRCTTACCCTGRGSAGAT		
		R2- 4163	GGTGTCASCMCCTATACGTCYCAT		
Mycobacterium spp.	rpoB	MycoF	GGCAAGGTCACCCCGAAGGG	764 bp, Tm: 64°C	Adékombi et al. 2003
		MycoR	AGCGGCTGCTGGGTGATCATC		

References

Adékambi T, Colson P, Drancourt M. rpoB-based identification of nonpigmented and late-pigmenting rapidly growing mycobacteria. J Clin Microbiol. 2003 Dec;41(12):5699-708. doi: 10.1128/jcm.41.12.5699-5708.2003. PMID: 14662964; PMCID: PMC308974.

Ahmed, N., Devi, S. M., Valverde, M., Vijayachari, P., Machang'u, R. S., Ellis, W. A., & Hartskeerl, R. A. (2006). Multilocus sequence typing method for identification and genotypic classification of pathogenic *Leptospira* species. Annals of clinical microbiology and antimicrobials, 5, 28. https://doi.org/10.1186/1476-0711-5-28

Andreani, J., Arnault, J. P., Bou Khalil, J. Y., Abrahão, J., Tomei, E., Vial, E., Le Bideau, M., Raoult, D., & La Scola, B. (2019). Atypical Cowpox Virus Infection in Smallpox-Vaccinated Patient, France. Emerging infectious diseases, 25(2), 212–219. https://doi.org/10.3201/eid2502.171433

Bassene, H., Sambou, M., Fenollar, F., Clarke, S., Djiba, S., Mourembou, G., LY A.B., Raoult D., Mediannikov, O. (2015). High prevalence of *Mansonella perstans* filariasis in rural Senegal. *The American Journal of Tropical Medicine and Hygiene*, 93: 601–606.

Benschop, K., Minnaar, R., Koen, G., van Eijk, H., Dijkman, K., Westerhuis, B., Molenkamp, R., Wolthers, K. (2010). Detection of human enterovirus and human parechovirus (HPeV) genotypes from clinical stool samples: polymerase chain reaction and direct molecular typing, culture characteristics, and serotyping. *Diagnostic Microbiology and Infectious Disease, 68(2), 166–173.*

Bouvresse, S., Socolovshi, C., Berdjane, Z., Durand, R., Izri, A., Raoult D., Chosidow, O. and Brouqui P. (2011). No evidence of *Bartonella quintana* but detection of *Acinetobacter baumannii* in head lice from elementary schoolchildren in Paris. *Comparative Immunology, Microbiology and Infectious Diseases*, 34:475–7.

Bruijnesteijn Van Coppenraet, E. S., Lindeboom, J. A., Prins, J. M., Peeters, M. F., Claas, E. C., & Kuijper, E. J. (2004). Real-time PCR assay using fine-needle aspirates and tissue biopsy specimens for rapid diagnosis of mycobacterial lymphadenitis in children. Journal of clinical microbiology, 42(6), 2644–2650. https://doi.org/10.1128/JCM.42.6.2644-2650.2004

Cohen-Bacrie, S.; Ninove, L.; Nougairède, A.; Charrel, R.; Richet, H.; Minodier, P. ; et al. (2011). Revolutionizing clinical microbiology laboratory organization in hospitals with in situ Point-of-Care. *PLoS ONE*, 6(7), e22403.

Corman et al. (2020). Diagnostic detection of Wuhan coronavirus 2019 by real-time RTPCR

Dahmani, M., Davoust, B., Benterki, M.S., Fenollar, F., Raoult, D., Mediannikov, O. (2015). Development of a new PCR-based assay to detect *Anaplasmataceae* and the first report of *Anaplasma phagocytophilum* and *Anaplasma platys* in cattle from Algeria. *Comparative Immunology, Microbiology & Infectious Diseases*, 39:39–45.

DeGraves, F. J., Gao, D., Hehnen, H. R., Schlapp, T., & Kaltenboeck, B. (2003). Quantitative detection of *Chlamydia psittaci* and *C. pecorum* by high-sensitivity real-time PCR reveals high prevalence of vaginal infection in cattle. Journal of clinical microbiology, 41(4), 1726–1729. https://doi.org/10.1128/jcm.41.4.1726-1729.2003

Edouard S, Tamalet C, Tissot-Dupont H, Colson P, Ménard A, Ravaux I, Dhiver C, Tomei C, Stein A, Raoult D. (2017). Evaluation of self-collected rectal swabs for the detection of bacteria responsible for sexually transmitted infections in a cohort of HIV-1-infected patients. *J Med Microbiol*. 66(6):693-697. doi: 10.1099/jmm.0.000481. PMID: 28590237.

Fall, N.S., Sarr, M., Diagne, N. et al. *Listeria monocytogenes* detected in vaginal self-samples of 2 women after spontaneous miscarriage, Senegal, West Africa. Eur J Clin Microbiol Infect Dis 39, 393–394 (2020). https://doi.org/10.1007/s10096-019-03739-0

Fenollar F., et al. (2008). Brevet: Method for diagnosis of and following a bacterial vaginosis by molecular quantification. *WO* 2008/062136 A2.

Fenollar, F., Mediannikov, O., Socolovschi, C., Bassene, H., Diatta, G., Richet, H., Tall, A., Sokhna, C., Trape, J.F and Raoult, D. (2010). *Tropheryma whipplei* bacteremia during fever in rural West Africa. *Clinical Infectious Diseases*, 51:515–521.

Folmer, O.; Black, M.; Hoeh, W.; Lutz, R.; Vrijenhoek, R. (1994). DNA primers for amplification of mitochondrial Cytochrome C oxidase subunit I from diverse metazoan invertebrates. Mol. Mar. Biol. Biotechnol., 3, 294–299.

Garcés-Sanchez, G., Wilderer, P.A., Munch, J.C., Horn, H. and Lebuhn, M. (2009). Evaluation of two methods for quantification of hsp70 mRNA from the waterborne pathogen *Cryptosporidium parvum* by reverse transcription real-time PCR in environmental samples. *Water Research*, 43: 2669–2678.

Ghai, R. R., Chapman, C. A., Omeja, P. A., Davies, T. J., & Goldberg, T. L. (2014). Nodule worm infection in humans and wild primates in Uganda: cryptic species in a newly identified region of human transmission. *PLoS neglected tropical diseases*, 8(1), e2641. https://doi.org/10.1371/journal.pntd.0002641

Greig, D.R., Hickey, T.J., Boxall, M.D, Begum, H., Gentle, A., Jenkins, C. and Chattaway, M. A., (2018). A real-time multiplex PCR for the identification and typing of Vibrio cholerae. *Diagnostic Microbiology and Infectious Disease*, 90: 171–176.

Heim, A., Ebnet, C., Harste, G. and Pring-Akerblom, P. (2003). Rapid and Quantitative Detection of Human Adenovirus DNA by Real-Time PCR. Journal of Medical Virology 70 :228–239.

Hopkins MJ, Ashton LJ, Alloba F, Alawattegama A, Hart IJ. (2010). Validation of a laboratory-developed real-time PCR protocol for detection of Chlamydia trachomatis and Neisseria gonorrhoeae in urine. Sex Transm Infect.86(3):207-11. doi: 10.1136/sti.2009.040634. PMID: 20522633.

Höhne, M., and Schreier, E. (2004). Detection and characterization of norovirus outbreaks in Germany: application of a one-tube RT-PCR using a fluorogenic real-time detectionsystem. *Journal of Medical Virology*, 72(2):312-9.

Izopet, J., Dubois, M., Bertagnoli, S., Lhomme, S., Marchandeau, S., Boucher, S., Kamar, N., Abravanel, F. and GuérinJ-L. (2012). Hepatitis E Virus Strains in Rabbits and Evidence of a Closely Related Strain in Humans, France. *Emerging Infectious Diseases*, 18(8): 1274–1281.

Jauregui LH, Higgins J, Zarlenga D, Dubey JP, Lunney JK. (2001). Development of a real-time PCR assay for detection of Toxoplasma gondii in pig and mouse tissues. J Clin Microbiol.;39(6):2065-71. doi: 10.1128/JCM.39.6.2065-2071.2001. PMID: 11376036; PMCID: PMC88090.

Keita ML, Medkour H, Sambou M, Dahmana H, Mediannikov O. (2020). Tabanids as possible pathogen vectors in Senegal (West Africa). *Parasit Vectors*. 1;13(1):500. doi: 10.1186/s13071-020-04375-w. PMID: 33004069; PMCID: PMC7528383.

Kessler, H.H., Mühlbauer, G., Rinner, B., Stelzl, E., Berger, A., Dörr, H.W., Santner, B., Marth, E. and Rabenau H. (2000). Detection of Herpes simplex virus DNA by real-time PCR. *Journal of Clinical* Microbiology, 38(7):2638-42.

Laidoudi Y, Ringot D, Watier-Grillot S, Davoust B, Mediannikov O. (2019). A cardiac and subcutaneous canine dirofilariosis outbreak in a kennel in central France. Parasite.;26:72. doi: 10.1051/parasite/2019073. Epub 2019 Dec 16. PMID: 31840652; PMCID: PMC6913249.

Laidoudi, Y., Davoust, B., Varloud, M., Niang, E.H. A., Fenollar, F. and Mediannikov, O. Development of a multiplexed qPCRs-based approach for the diagnosis of *Dirofilaria immitis, D. repens, Acanthocheilonema reconditum* and the others filariosis. bioRxiv 842575; doi: https://doi.org/10.1101/842575.

Laidoudi, Y., Medkour, H., Latrofa, M. S., Davoust, B., Diatta, G., Sokhna, C., Barciela, A., Hernandez-Aguilar, R. A., Raoult, D., Otranto, D., & Mediannikov, O. (2020). Zoonotic *Abbreviata caucasica* in Wild Chimpanzees (*Pan troglodytes verus*) from Senegal. Pathogens (Basel, Switzerland), 9(7), 517. https://doi.org/10.3390/pathogens9070517

Laidoudi, Y., Medkour, H., Levasseur, A., Davoust, B., & Mediannikov, O. (2020). New Molecular Data on *Filaria* and its *Wolbachia* from Red Howler Monkeys (*Alouatta macconnelli*) in French Guiana-A Preliminary Study. *Pathogens* (Basel, Switzerland), 9(8), 626. https://doi.org/10.3390/pathogens9080626

Laidoudi, Y., Ringot, D., Watier-Grillot, S., Davoust, B., Mediannikov O. (2019). A cardiac and subcutaneous canine dirofilariosis outbreak in a kennel in central France. *Parasite*, 26: 72. doi:10.1051/parasite/2019073.

Leslie, D. E., Azzato, F., Karapanagiotidis, T., Leydon, J., & Fyfe, J. (2007). Development of a real-time PCR assay to detect Treponema pallidum in clinical specimens and assessment of the assay's performance by comparison with serological testing. Journal of clinical microbiology, 45(1), 93–96. https://doi.org/10.1128/JCM.01578-06

Liu, J., Gratz, J., Amour, C., Kibiki, G., Becker, S., Janaki, L., Verweij, J.J., Taniuchi, M., Sobuz, S.U., Haque, R., Haverstick, D.M. and Houpt E.R. (2013). A laboratory-developed taqman array card for simultaneous detection of 19 enteropathogens. *Journal of Clinical Microbiology*, 2013;51: 472–480.

Logan, C., O'Leary, J.J. and O'Sullivan, N. (2007). Real-time Reverse Transcription PCR Detection of Norovirus, Sapovirus and Astrovirus as Causative Agents of Acute Viral Gastroenteritis. *Journal of Virological Methods*, 146(1-2):36-44.

Luna, R. A., Boyanton, B. L., Jr, Mehta, S., Courtney, E. M., Webb, C. R., Revell, P. A., & Versalovic, J. (2011). Rapid stool-based diagnosis of Clostridium difficile infection by real-time PCR in a children's hospital. *Journal of clinical microbiology*, 49(3), 851–857. https://doi.org/10.1128/JCM.01983-10

Ly, T., Kerbaj, J., Edouard, S., Hoang, V. T., Louni, M., Dao, T. L., Benkouiten, S., Badiaga, S., Tissot-Dupont, H., Raoult, D., Brouqui, P., Mediannikov, O., & Gautret, P. (2019). The Presence of Acinetobacter baumannii DNA on the Skin of Homeless People and Its Relationship With Body Lice Infestation. Preliminary Results. Frontiers in cellular and infection microbiology, 9, 86. https://doi.org/10.3389/fcimb.2019.00086

Manara, S., Asnicar, F., Beghini, F., Bazzani, D., Cumbo, F., Zolfo, M., Nigro, E., Karcher, N., Manghi, P., Metzger, M.I., Pasolli, E. and Segata, N. (2019). Microbial Genomes From Non-Human Primate Gut Metagenomes Expand the Primate-Associated Bacterial Tree of Life With Over 1000 Novel Species. *Genome Biology*, 20 : 299. doi: 10.1186/s13059-019-1923-9.

Mediannikov, O., Fenollar, F., Socolovschi, C., Diatta, G., Bassene, H., Molez, J-F., Cheikh Sokhna, C., Trape, J-F and Raoult D. (2010). *Coxiella burnetii* in humans and ticks in rural Senegal. PLOS Neglected Tropical Diseases 4: e654.

Medkour H, Amona I, Laidoudi Y, Davoust B, Bitam I, Levasseur A, Akiana J, Diatta G, Pacheco L, Gorsane S, Sokhna C, Hernandez-Aguilar RA, Barciela A, Fenollar F, Raoult D, Mediannikov O. (2020). Parasitic Infections in African Humans and Non-Human Primates. *Pathogens.*;9(7):561. doi: 10.3390/pathogens9070561. PMID: 32664573; PMCID: PMC7400533.

Medkour, H., Laidoudi, Y., Athias, E., Bouam, A., Dizoé, S., Davoust, B. and Mediannikov, O. (2020). Molecular and serological detection of animal and human vector-borne pathogens in the blood of dogs from Côte d'Ivoire. *Comparaive Immunology Microbiology Infectious Diseases,* 69: 101412. doi: 10.1016/j.cimid.2019.101412.

Medkour, H., Varloud, M., Davoust, B., Mediannikov, O. (2020). New Molecular Approach for the Detection of *Kinetoplastida* Parasites of Medical and Veterinary Interest. *Microorganisms*, 8: 356. doi:10.3390/microorganisms8030356.

Menard, A., Santos, A., Megraud, F. and Oleastro M. (2002). PCR-restriction fragment length polymorphism can also detect point mutation A2142C in the 23S rRNA gene, associated with Helicobacter pylori resistance to clarithromycin. *Antimicrob Agents Chemother* 46:1156–7.

Mombo, I.M., Berthet, N., Lukashev, A.N., Bleicker, T., Brünink, S., Berthet, N., Maganga G.D., Durand, P., Arnathau, C. Boundenga, L., Ngoubangoye, B., Boué B., Liégeois F., Ollomo B., Prugnolle F., Drexler, J.F., Drosten, C., Renaud, F., Rougeron, V., and Leroy, E. (2017). African Non-Human Primates Host Diverse Enteroviruses. *PLOS ONE,* 12(1), e0169067. doi: 10.1371/journal.pone.0169067.

Monjure, C. J., Tatum, C. D., Panganiban, A. T., Arainga, M., Traina-Dorge, V., Marx, P. A., Jr, & Didier, E. S. (2014). Optimization of PCR for quantification of simian immunodeficiency virus genomic RNA in plasma of rhesus macaques (*Macaca mulatta*) using armored RNA. Journal of medical primatology, 43(1), 31–43. https://doi.org/10.1111/jmp.12088

Motte, A., Blanc, J., Minodier, P.,and Colson, P. (2009). Acute hepatitis A in a pregnant woman at delivery. *International Journal of Infectious Diseases,* 13(2), e49–e51.

Mourembou, G., Fenollar, F., Socolovschi, C., Lemamy, G.J., Nzoughe, H., Kouna, L.C., Toure-Ndouo, F., Million, M., Mbiguino, A.N., Lekana-Douki, J.B., Raoult, D. (2015). Molecular detection of fastidious and common bacteria as well as *Plasmodium* spp. in febrile and afebrile children in Franceville, Gabon. *The American Journal of Tropical Medicine and Hygiene,* 92:926–932.

Mourembou, G., Lekana-Douki, J.B, Mediannikov, O., Maghendji Nzondo, S., Kouna, L.C, Biteghe Bi Essone, J.C., Fenollar, F., Raoult, D. (2015). Possible Role of Rickettsia felis in Acute Febrile Illness among Children in Gabon. *Emerging Infectious Diseases*, 21(10): 1808–1815.

Ninove, L., Nougairede, A., Gazin, C., Thirion, L., Delogu, I., Zandotti, C., Charrel, R. N., & De Lamballerie, X. (2011). RNA and DNA bacteriophages as molecular diagnosis controls in clinical virology: a comprehensive study of more than 45,000 routine PCR tests. PloS one, 6(2), e16142. https://doi.org/10.1371/journal.pone.0016142

Parola, P., Diatta, G., Socolovschi, C., Mediannikov, O., Tall, A., Bassene, H., Trape, J.F. and Raoult D., (2011). Tick-borne relapsing Fever borreliosis, rural Senegal. *Emerging Infectious Diseases,* 17(5): 883–885.

Praet, N., Verweij, J.J., Mwape, K.E, Phiri, I.K, Muma, J.B., Zulu, G., van Lieshout, L., Rodriguez-Hidalgo, R., Benitez-Ortiz, W., Dorny P., Gabriël, S. (2013). Bayesian modelling to estimate the test characteristics of coprology, coproantigen ELISA and a novel real-time PCR for the diagnosis of taeniasis. *Tropical Medicine* and *International Health,*18: 608–614.

Riehm JM, Rahalison L, Scholz HC, Thoma B, Pfeffer M, Razanakoto LM, Al Dahouk S, Neubauer H, Tomaso H. (2011). Detection of Yersinia pestis using real-time PCR in patients with suspected bubonic plague. Mol Cell Probes. 25(1):8-12. doi: 10.1016/j.mcp.2010.09.002. Epub 2010 Oct 8. PMID: 20933595.

Roy S., Vandenberghe L.H., Kryazhimskiy S., Grant R., Calcedo R., Yuan X., Keough M., Sandhu A., Wang Q., Medina-Jaszek C.A., et al. (2009). Isolation and characterization of adenoviruses persistently shed from the gastrointestinal tract of non-human primates. *PLoS Pathog.* 5:1–9. doi: 10.1371/journal.ppat.1000503.

Roy, S., Kabir, M., Mondal, D., Ali, I.K.M., Petri, W.A., Haque R. (2005). Real-time-PCR assay for diagnosis of *Entamoeba histolytica* infection. *Journal of Clinical Microbiology*, 43: 2168–2172.

Smythe, L. D., Smith, I. L., Smith, G. A., Dohnt, M. F., Symonds, M. L., Barnett, L. J., and McKay, D. B. (2002). A quantitative PCR (TaqMan) assay for pathogenic Leptospira spp. *BMC infectious diseases*, 2, 13. https://doi.org/10.1186/1471-2334-2-13.

Salazar JC, Rathi A, Michael NL, Radolf JD, Jagodzinski LL. (2007). Assessment of the kinetics of Treponema pallidum dissemination into blood and tissues in experimental syphilis by real-time quantitative PCR. *Infect Immun.* 75(6):2954-8. doi: 10.1128/IAI.00090-07. Epub 2007 Apr 16. PMID: 17438037; PMCID: PMC1932886.

Sow, D., Parola, P., Sylla, K., Ndiaye, M., Delaunay, P., Halfon, P., Camiade, S., Dieng, T., Tine, R.C.K., Faye, B., Ndiaye, J.L., Dieng, Y., Gaye, O., Raoult, D., Bittar F. (2017). Performance of Real-Time Polymerase Chain Reaction Assays for the Detection of 20 Gastrointestinal Parasites in Clinical Samples from Senegal. *American Journal* of *Tropical Medicine* and *Hygiene,* 97(1):173-182.

Tamalet C, Richet H, Carcopino X, et al. Testing for human papillomavirus and measurement of viral load of HPV 16 and 18 in self-collected vaginal swabs of women who do not undergo cervical cytological screening in Southern France. J Med Virol. 2010;82(8):1431-1437. doi:10.1002/jmv.21835

Tapparel, C., Cordey, S., Van Belle, S., Turin, L., Lee, W.M., Regamey, N., Meylan, P., Mühlemann, K., Gobbini, F. and Kaiser, L.(2009) New Molecular Detection Tools Adapted to Emerging Rhinoviruses and Enteroviruses. Journal Of Clinical Microbiology, 47(6): 1742–1749.

Touré, F.S., Bain, O., Nerrienet, E., Millet, P., Wahl, G., Toure, Y., Doumbo, O., Nicolas, L. , Georges, A.J., McReynolds, L.A., Egwang, T.G . (1997). Detection of *Loa loa* specific DNA in blood from occult-infected individuals. Experimental *Parasitology,* 1997;86: 163–170.

Verweij, J.J., Canales, M., Polman, K., Ziem, J., Brienen, E.A., Polderman, A.M., van Lieshout L. (2009). Molecular diagnosis of *Strongyloides stercoralis* in faecal samples using real-time PCR. *Transactions of the Royal Society of Tropical Medicine and Hygiene,* 103: 342–346.

Verweij, J.J., Blange, R.A., Templeton, K., Schinkel, J., Brienen, E.A.T., Rooyen, M.A.A. van Rooyen, van Lieshout, L. and Polderman, A. M. (2004). Simultaneous Detection of *Entamoeba histolytica*, *Giardia lamblia*, and *Cryptosporidium parvum* in Fecal Samples by Using Multiplex Real-Time PCR. *Journal of Clinical Microbiology,* 42: 1220–1223.

Verweij, J.J., Brienen, E.A.T., Ziem, J., Yelifari, L., Polderman, A.M., Van Lieshout, L. (2007). Simultaneous detection and quantification of *Ancylostoma duodenale, Necator americanus,* and *Oesophagostomum bifurcum* in fecal samples using multiplex real-time PCR. *American Journal* of *Tropical Medicine* and *Hygiene*, 77: 685–690.

Verweij, J.J., Laeijendecker, D., Brienen E.A.T., Van Lieshout, L. and Polderman, A.M. (2003). Detection of *Cyclospora cayetanensis* in travellers returning from the tropics and subtropics using microscopy and real-time PCR. *International Journal of Medical Microbiology,* 293: 199–202.

Wiria A.E., Prasetyani, M.A., Hamid, F., Wammes, L.J., Lell, B., Ariawan, I., Uh, H.W., Wibowo, H., Djuardi, Y., Wahyuni, S., Sutanto, I., May, L., Luty, A.J., Verweij, J.J., Sartono, E., Yazdanbakhsh, M., Supali, T. (2010). Does treatment of intestinal helminth infections influence malaria? Background and methodology of a longitudinal study of clinical, parasitological and immunological parameters in Nangapanda, Flores, Indonesia (Immuno SPIN Study). *BMC Infectious Diseases*, 10:77. doi: 10.1186/1471-2334-10-77.

Zeng, S.Q., Halkosalo, A., Salminen, M., Szakal, E.D., Puustinen, L., Vesikari, T. (2008). One-step quantitative RT-PCR for the detection of rotavirus in acute Gastroenteritis. *Journal of Virological Methods*, 153 (2008) 238–240.

Supplementary material S1. Sequences used for primer design.

A. Sequences used for design of primers for *Treponema* spp.:

NR_076531.1_Treponema_pallidum_subsp._pallidum_strain_SS14;
JX120546.1_Treponema_paraluiscuniculi_strain_Cuniculi_A ; NR_076899.1_Treponema_caldarium_strain_H1 ;
NR_076709.1_Treponema_primitia_strain_ZAS-2 ; NR_076878.1_Treponema_brennaborense_strain_DD5/3 ;
NR_076766.1_Treponema_paraluiscuniculi_strain_Cuniculi_A ; NR_076212.1_Treponema_denticola_strain_ATCC_35405;
NR_076708.1_Treponema_azotonutricium_strain_ZAS-9; NR_103187.1_Borrelia_hermsii_HS1_strain_DAH;
NR_103185.1_Borrelia_turicatae; NR_076567.1_Borrelia_recurrentis_strain_A1;
NR_103181.1_Borrelia_crocidurae_strain_Achema; NR_103969.1_Borrelia_burgdorferi;
NR_103964.1_Leptospira_biflexa_serovar_Patoc_strain_Patoc_1;
NR_076199.1_Leptospira_interrogans_serovar_Copenhageni_strain_Fiocruz_L1-130; X14249.1_Leptospira_interrogans;
NR_103021.1_Brachyspira_intermedia_strain_PWS/A; NR_076731.1_Brachyspira_murdochii_strain_56-150;
NR_076624.1_Brachyspira_hyodysenteriae_strain_WA1

B. Sequences used for design of primers for *EMCV*:

KM269482.1_ Encephalomyocarditis_virus_strain_ATCC_VR-129B;
JQ864080.1_Encephalomyocarditis_virus_isolate_HB10 ; GU181317_strain_SPU_64/03_Bonobo_RDC ;
DQ835185_strain_1086C_Rattus_Belgium;
AJ235731.1_Encephalomyocarditis_virus_mRNA_for_3D_pigs_rodents_in_Europe;
AJ235728.1_EEncephalomyocarditis_virus_mRNA_for_3D_pigs_rodents_in_Europe;
DQ294633.1_Mengo_virus_isolate_in_cell_culture; L22089.1_Mengo_virus_isolate_M;
DQ835185.2_Encephalomyocarditis_virus_strain_1086C; KU955338.1_Mengo_virus_strain_AnrB-3741;
AJ235733.1_Encephalomyocarditis_virus_mRNA_for_3Dpol_protein_strain_ITL-135/86;
AY296731.1_Encephalomyocarditis_virus_EMCV-30; X87335.1_Encephalomyocarditis_virus;
M37588.1_Encephalomyocarditis_virus_diabetogenic_variant_(D_variant_ifp-_phenotype);
M22457.1_Encephalomyocarditis_(EMC)_virus_EMC-B; KC310738.1_Encephalomyocarditis_virus_isolate_Sing-M105-02; KC310737.1_Encephalomyocarditis_virus_isolate_Sing-M100-02;

C. Sequences used for design of primers for *Leptospira* spp.:

As the same manner, we used sequences available in GenBank databases of *Leptospira* spp., and we designed primers targeting all of 16S, 23S, LipL 32, LipL 41, LipL 71 and YidC genes.
